# nCas9 Engineering for Improved Target Interaction Presents an Effective Strategy to Enhance Base Editing

**DOI:** 10.1002/advs.202405426

**Published:** 2024-06-17

**Authors:** Guiquan Zhang, Ziguo Song, Shisheng Huang, Yafeng Wang, Jiayuan Sun, Lu Qiao, Guanglei Li, Yuanyuan Feng, Wei Han, Jin Tang, Yulin Chen, Xingxu Huang, Furui Liu, Xiaolong Wang, Jianghuai Liu

**Affiliations:** ^1^ Zhejiang Lab Hangzhou Zhejiang 311121 China; ^2^ International Joint Agriculture Research Center for Animal Bio‐Breeding Ministry of Agriculture and Rural Affairs/Key Laboratory of Animal Genetics Breeding and Reproduction of Shaanxi Province College of Animal Science and Technology Northwest A&F University Yangling Shaanxi 712100 China; ^3^ Department of Rheumatology and Immunology Nanjing Drum Tower Hospital, Affiliated Hospital of Medical School Nanjing University Nanjing 210008 China; ^4^ Gene Editing Center School of Life Science and Technology ShanghaiTech University 100 Haike Rd., Pudong New Area Shanghai 201210 China; ^5^ State Key Laboratory of Pharmaceutical Biotechnology and MOE Key Laboratory of Model Animals for Disease Study Model Animal Research Center at Medical School of Nanjing University Nanjing 210061 China

**Keywords:** base editor, enhanced BE, nCas9 engineering, non‐targeted strand, *T*‐cells

## Abstract

Base editors (BEs) are a recent generation of genome editing tools that couple a cytidine or adenosine deaminase activity to a catalytically impaired Cas9 moiety (nCas9) to enable specific base conversions at the targeted genomic loci. Given their strong application potential, BEs are under active developments toward greater levels of efficiency and safety. Here, a previously overlooked nCas9‐centric strategy is explored for enhancement of BE. Based on a cytosine BE (CBE), 20 point mutations associated with nCas9‐target interaction are tested. Subsequently, from the initial positive X‐to‐arginine hits, combinatorial modifications are applied to establish further enhanced CBE variants (1.1–1.3). Parallel nCas9 modifications in other versions of CBEs including A3A‐Y130F‐BE4max, YEE‐BE4max, CGBE, and split‐AncBE4max, as well as in the context of two adenine BEs (ABE), likewise enhance their respective activities. The same strategy also substantially improves the efficiencies of high‐fidelity nCas9/BEs. Further evidence confirms that the stabilization of nCas9‐substrate interactions underlies the enhanced BE activities. In support of their translational potential, the engineered CBE and ABE variants respectively enable 82% and 25% higher rates of editing than the controls in primary human *T*‐cells. This study thus demonstrates a highly adaptable strategy for enhancing BE, and for optimizing other forms of Cas9‐derived tools.

## Graphical abstract

This study explores a previously overlooked nCas9‐centric strategy for enhancement of base editors (BE). Structure‐guided protein engineering establishes nCas9 variants that can commonly enhance a collection of BE tools. In support of their translational potential, the engineered CBE and ABE variants respectively enable 82% and 25% higher rates of editing than the controls in primary human *T*‐cells.

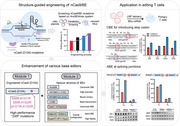

## 1 Introduction

The development of base editors (BEs) represents a significant breakthrough toward the goal of enabling precise and safe genome editing. In principle, the base editors are constructed by the fusion of a catalytically impaired CRISPR‐Cas nuclease (D10A) with a single‐stranded DNA‐selective deaminase domain.^[^ ^1^, ^2^ ^]^ BE action is initiated by sgRNA‐guided target DNA binding.^[^ ^1^ ^]^ This leads to the formation of an R‐loop structure containing a DNA:RNA hybrid and a displaced, single‐stranded DNA (ssDNA) segment.^[^ ^3^ ^]^ Certain suitably positioned cytosine(s) or adenine(s) in the ssDNA loop of the non‐targeted strand (NTS) are subsequently subjected to catalysis by the particular deaminase domain, triggering subsequent DNA repair events which in turn lead to programmed base changes on both strands. Such innovative design allows the introduction of specific point mutations, while circumventing the requirement of introducing DNA double‐strand breaks (DSBs) and co‐administrating a repair template.^[^ ^1^, ^2^, ^4^ ^]^

The initial version of BE harnesses the cytidine deaminase activity of the APOBEC enzyme family (fused to a nuclease‐dead Cas9 moiety] to enable C‐to‐T edits (CBE).^[^ ^1^ ^]^ At the specified site, targeted deamination of cytosine base yields uracil which instead features a thymine‐equivalent base‐pairing property. To inhibit the cellular base excision repair for prematurely removing the uracil base, the CBE also incorporates a module of uracil glycosylase inhibitor (UGI). In addition, the adoption of a D10A Cas9 nickase (nCas9) for single‐stranded nicking of the sgRNA‐complemented strand can instruct the DNA repair system to drive coordinated C:G‐to‐T:A base changes on both strands. Subsequent to the initial development, many cytidine deaminase domains have been adopted to construct base editors with various editing windows, sequence context preferences, and base‐converting efficiencies.^[^ ^4^ ^]^ Other cytosine‐editing platforms have instead harnessed the base excision repair to enable cytosine conversion to non‐T bases upon deamination. For instance, the incorporation of uracil‐DNA glycosylase in lieu of UGI in a cytidine base editor was found to stimulate uracil removal and the preferential installation of G in the subsequent repair (CGBE).^[^ ^5^ ^]^ In parallel to the cytosine base editors, the adenine base editors (ABEs) have also been developed.^[^ ^2^ ^]^ Therein, the adoption of a laboratory‐evolved adenosine deaminase that produces inosine in DNA substrates drives the subsequent A:T‐to‐G:C conversions at the target sites.

The potential for genome editing tools hinges heavily on their efficiencies. In this regard, a number of investigations have sought to enhance base editing efficiencies. In general, the optimization of deaminase activities and the overall BE architecture has led to the establishment of various versions of BEs with improved activities.^[^ ^6^, ^7^ ^]^ In addition, it has also been reported that the introduction of single‐stranded DNA binding domain of the Rad51 protein to stabilize the R‐loop structure promotes BE efficiencies and expands the editing window.^[^ ^8^ ^]^ It is worth noting that compared with the recent version of highly active ABE (i.e., ABE8e),^[^ ^9^ ^]^ the current cytosine‐converting BEs are generally less active.^[^ ^10^ ^]^ Exploration of additional strategies may be warranted for further improvements of cytosine base editors, and for realizing the application potential of BEs in general. Moreover, the application of BE is associated with off‐target base editing at both Cas‐dependent and Cas‐independent sites, and at numerous variable sites in cellular RNAs.^[^ ^4^ ^]^ Therefore, improvement of base editing precision represents another key consideration for BE developments.

Here, to enhance base editors, we shift the focus to the nCas9 moiety and employ rational mutagenesis toward its improved competence of target DNA binding. Interestingly, analogous strategies have served to increase the potencies of several engineered Cas9 specialty‐variants (e.g., for PAM relaxation),^[^ ^11^, ^12^, ^13^ ^]^ as well as those of other less active Cas proteins (e.g., Cas12a and Cas12f).^[^ ^14^, ^15^, ^16^, ^17^ ^]^ It is also conceivable that a nCas9‐oriented optimization approach would be compatible with various CBE/ABE forms, while spared from exacerbating the risk of spurious, Cas‐independent non‐specific base editing.^[^ ^4^ ^]^ In addition, the same modifications could be introduced to high‐fidelity nCas9 moieties,^[^ ^18^, ^19^ ^]^ to offset their reduced potencies and to enable effective high‐fidelity base editing.^[^ ^20^ ^]^ Based on previous structure models on the interaction of SpCas9/sgRNA with the DNA substrate,^[^ ^3^, ^18^ ^]^ we test a collection of corresponding nCas9 variants in the architecture of AncBE4max. We then establish specifically engineered nCas9 variants that contribute to higher base‐editing activity in various BE tools. Our work puts forward a highly adaptable and effective strategy to enhance BEs to aid their future developments and applications.

## 2 Results

### 2.1 Structure‐Guided Engineering of nCas9 to Increase AncBE4max Editing Efficiencies

**Figure 1:**
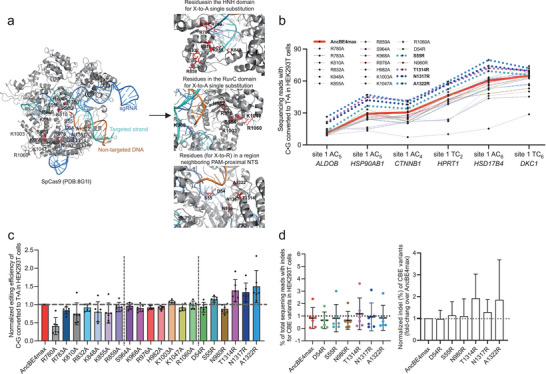
Structure‐guided engineering of nCas9 to increase AncBE4max editing efficiencies. a) On the left, the positions of individual point mutations tested are shown on the solved structure of SpCas9 in complex with sgRNA and target DNA (PDB: 8G1I). The locations of the focused amino acids are highlighted in red color with black letter labels. On the right, the zoomed‐in structures show part of the HNH domain, RuvC domain, and a region near the initially unwound non‐targeted strand. The latter contains the amino acid residues where individual X‐to‐R substitutions were made. b) Parallel comparison of editing activities by AncBE4max and 20 single‐substitution, engineered nCas9/AncBE4max variants at six genomic loci in HEK293T cells. The results show the percentage of C‐to‐T conversion at the mainly edited position within the editing window. The AncBE4max and four single substitution groups (S55R, T1314R, N1317R, and A1322R) with increased editing efficiencies are highlighted in bold. Data are presented as means ± s.d. (*n* = 3 biological replications). c) Results in (b) were further analyzed by considering editing efficiency at all sites (means ± s.d., *n* = 6 sites) as a whole. The editing levels induced by the control AncBE4max at each site were set as 1 (position marked by horizontal gray dashed line). d) The percentages of editing‐associated indels at six target sites by the six X‐to‐R variants are shown (see legend of (a, b) for reference). The left graph corresponds to the true indel percentages (means ± s.d.). The indel levels normalized to those by AncBE4max are shown on the right (error bar: s.d.).

BEs are recruited to specific DNA targets via the nCas9 moiety in complex with the sgRNA.^[^ ^1^, ^2^ ^]^ We sought to investigate whether mutations affecting nCas9‐target interaction would also impact BE activities. A popular version of AncBE4max editor was used as the prototype.^[^ ^6^ ^]^ We began by inquiring into the HNH and RuvC nuclease domains of Cas9 (nCas9) that apparently interact with the target DNA. Previous studies have proposed a number of amino acid residues (mainly the positively charged arginine (R) and lysine (K) residues) in the HNH and RuvC nuclease domains of Cas9 for potentially shaping the interactions with the target strand (TS), and with the opposite NTS of the substrate DNA, respectively.^[^ ^3^, ^18^ ^]^ In accordance, we decided to introduce individual alanine (A) substitution of such residues (with respectively 7 residues in the HNH and RuvC domains) to the nCas9 moiety of AncBE4max (**Figure** **1**a, using PDB: 8G1I to derive the structural model). Note that the present investigation did not encompass the helical recognition lobe (REC domains) that also make contact with the target DNA.^[^ ^21^ ^]^

To analyze the activities of the prototype AncBE4max or the modified variants, HEK293T cells were transfected with the BEs in combination with various sgRNAs to target six genomic loci. Forty‐eight hours following transfection of the editing plasmids, the transfectants were enriched and harvested with the aid of flow cytometry (selection for a fluorescent marker encoded by the sgRNA plasmid). The genomic DNA samples were subjected to next‐generation sequencing (NGS) analyses for the programmed C‐to‐T editing efficiencies. As adopted to be a field routine,^[^ ^1^ ^]^ the base corresponding to the first 5′ nucleotide of a 20‐bp sgRNA spacer is numbered as position “1”. For simplicity of data presentation, the editing efficiencies at the first substantially modified C (in a multiple‐C target) were used for quantitation (Figure 1b). Indeed, relative levels of editing at co‐situated Cs in the editing windows generally remained consistent across all variants tested herein (Figure S1, Supporting Information and see later sections).

Examinations of relative editing efficiencies of different X‐to‐A AncBE4max variants (X denoting the original amino acid residue) showed some interesting trends (Figure 1b,c). First, single‐substitution variants in the HNH domain‐subgroup (R780A, R783A, K810A, R832A, K848A, K855A and R859A) were often associated with visible decreases (≈10% to 60% for 6 out of 7 variants) of base editing efficiencies relative to the control levels (see Figure 1c for normalized levels). In comparison, the counterparts in the RuvC domain‐subgroup (S964A, K968A, R976A, H982A, K1003A, K1047A, and R1060A) generally did not show changes in base editing activities (Figure 1b,c). Such contrasting patterns appeared to correlate with a role by the nuclease activity of the HNH domain, but not of the RuvC domain (within D10A nCas9), in AncBE4max base editing.^[^ ^1^ ^]^ It is therefore possible that the tested R/K residues in the HNH domain may contribute to its optimal nuclease activity, which in turn promote the installation of base edits. Some variants (HNH subgroup) may also present changes in conformational dynamics that impact on the cleavage fidelity control, as shown previously in the Cas9 variants harboring the K848A, K810A or K855A substitutions.^[^ ^18^, ^22^ ^]^ On the other hand, the general lack of effects by the tested single X‐to‐A RuvC domain mutants might point to the collective actions from these positively charged/polar residues for substrate binding.

In addition to the X‐to‐A variants, we also explored other nCas9/AncBE4max variants potentially bearing greater affinities to the DNA substrate. Such variants could be engineered by replacement of suitable non‐ or negatively‐charged residues into positively charged arginine residues (X‐to‐R).^[^ ^11^, ^14^, ^16^ ^]^ Given the potential fidelity complications that may be associated with HNH domain‐engineered variants,^[^ ^18^, ^22^ ^]^ we examined the interaction of the RuvC domain with the single‐stranded region in the NTS of DNA. Since upon Cas9/sgRNA targeting, the formation of ssDNA proceeds in a PAM‐proximal to PAM‐distal direction, the interaction of the initially unwound ssDNA with Cas9 would conceivably play a greater role in stabilizing the targeting complex. Previous study of the Cas9/sgRNA/DNA structure has strongly suggested the interaction of S55 residue in Cas9 with the initially unwound ssDNA backbone between nucleotides −2 and −3 upstream of the PAM motif.^[^ ^3^ ^]^ Therefore, respective X‐to‐R modifications were established on S55 of nCas9/AncBE4max, as well as on five other residues positioned at spatial proximity to the initially unwound ssDNA (D54, N980, T1314, N1317, A1322,^[^ ^13^ ^]^ the latter three located within a loop structure in C‐terminal domain] (Figure 1a).

Notably, compared to the original version of AncBE4max, moderate enhancements of C‐to‐T editing efficiencies were observed in four out of six such X‐to‐R variants (S55R, T1314R, N1317R, and A1322R) (Figure 1b,c; Figure S1, Supporting Information). On the other hand, the editing activities by D54R and N980R variants showed slight decreases, conveniently serving as intrinsic controls for the other X‐to‐R variants. Formal quantitation was made considering the averaged, relative editing efficiencies over the control levels at six loci (per‐site normalization). The averaged activities of S55R, T1314R, N1317R, and A1322R variants were respectively 1.1‐, 1.4‐, 1.3‐, and 1.5‐fold of the activity shown by the original AncBE4max (Figure 1c). It is also worth mentioning that at all sites tested, these variants consistently exhibited editing levels higher than those by the prototype AncBE4max (Figure 1b).

CBEs can induce low levels of on‐target indels which are the byproducts of cellular base excision repair (BER) engaged upon the initial cytosine‐to‐uracil conversion.^[^ ^23^ ^]^ Conceivably, similar to those of the desired base edits, the degrees of such editing impurity may also be shaped by nCas9‐DNA interactions. We confirmed that the overall incidences of indels (at 6 genomic loci) by AncBE4max were very low (with averages < 1%). The X‐to‐R variants featured moderately higher (S55R, T1314R, N1317R, and A1322R) or similar (D54R and N980R) indel rates relative to those of the original AncBE4max (Figure 1d). Although cautions need to be taken on interpreting such low‐frequency measurements, it is noteworthy that the overall patterns of indel rates (Figure 1d, right) and precise base edits (Figure 1c) by the control and X‐to‐R AncBE4max variants showed some resemblances. Indeed, a similar positive correlation between the levels of desirable edits and indels was observed previously when BEs with different deaminase domains were surveyed.^[^ ^7^ ^]^ Therefore, the results above further support the conclusion that S55R, T1314R, N1317R, and A1322R AncBE4max indeed present higher editing activities.

### 2.2 Combination of X‐to‐R Mutations for Further Improvement of AncBE4max Activities

**Figure 2:**
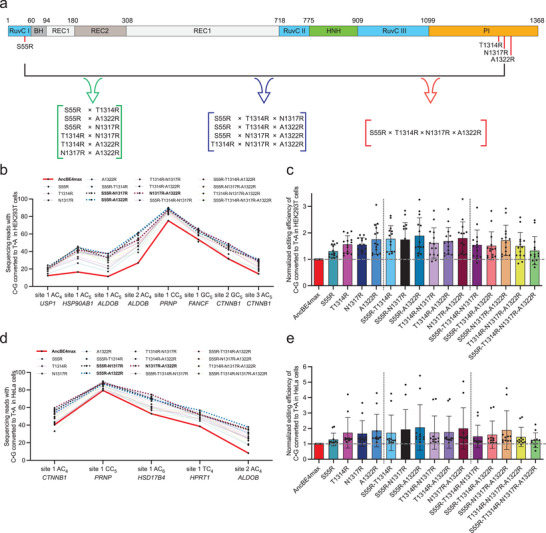
Combination of X‐to‐R mutations for further improvement of AncBE4max activities. a) The illustration shows the linear domain arrangement of Cas9, with the four enhancing, X‐to‐R substitution sites marked using red solid lines. The brackets in green, deep blue, and red represent double, triple, and quadruple combinatorial mutations, respectively. b) Parallel comparisons were made for AncBE4max, 4 single X‐to‐R variants and 11 combinatorial X‐to‐R variants at eight genomic loci in HEK293T cells. The results show the percentage of C‐to‐T conversion at the mainly edited position within the editing window. The red bold line highlights AncBE4max as the benchmark, and the three bold dashed lines mark the top‐performer double X‐to‐R variants. Data are presented as means ± s.d. (*n* = 3 biological replications). c) Results in (b) were further analyzed by considering editing efficiency at all sites (means ± s.d., *n* = 8 sites) as a whole. The editing levels induced by the control AncBE4max at each site were set as 1 (position marked by a horizontal gray dashed line). d) The experiment similar to (b) was carried out in HeLa cells at five endogenous genomic loci. The results show the percentage of C‐to‐T conversion at the mainly edited position within the editing window. The red bold line highlights AncBE4max as the benchmark, and the three bold dashed lines mark the top‐performer double X‐to‐R variants. Data are means ± s.d. (*n* = 3 biological replications). e) Results in (d) were further analyzed by considering editing efficiency at all sites (means ± s.d., *n* = 5 sites) as a whole. The editing levels induced by the control AncBE4max at each site were set as 1 (position marked by a horizontal gray dashed line).

Next, we asked whether various combination of these four point‐substitutions could further improve the base‐editing efficiencies of AncBE4max. To this end, we constructed AncBE4max variants harboring double, triple, and quadruple S55R/T1314R/N1317R/A1322R substitutions in the nCas9 moiety (with 6, 4 and 1 variants in each sub‐group) (**Figure** **2**a). These eleven Xs‐to‐Rs variants and the single X‐to‐R variants were tested first in HEK293T cells for editing efficiencies. We selected a total of eight different target sites within the genome, including two sites investigated earlier (see Figure 1) (Figure 2b; Figure S2a, Supporting Information). Cells were harvested 48 h after being transfected with the editing plasmids. Following amplification of the targeted sites by PCR, the products were analyzed by NGS. To assess the overall editing efficiencies by the AncBE4max variants (relative to the control level), all major C‐to‐T editing events detected at the target sites were considered (Figure 2c; Figure S2a, Supporting Information). Expectedly, the results from such an independent experiment further validated that all four single X‐to‐R variants presented higher editing efficiencies than the prototype AncBEmax4 (Figure 2b,c). The overall patterns of relative activities among these single X‐to‐R variants were also very similar to those shown earlier (see Figure 1c). Consistent with the positive effects by the individual X‐to‐R substitutions, all variants with combinatorial substitutions showed higher activities than the prototype AncBE4max (Figure 2b,c). In addition, it was noted that combinations of two X‐to‐R substitutions often resulted in a further improvement in efficiency over that by either of the parental variants (except for the T1314R‐N1317R variant) (Figure 2b,c; Figure S2a, Supporting Information). As an example, in this experiment, the S55R‐A1322R AncBE4max demonstrated the group‐best (among all variants) overall efficiencies, which on average represented a 1.9‐fold increase over the control level (Figure 2c). In reference, the S55R and A1322R variants respectively featured 1.3‐ and 1.7‐fold increases over the control level. When the actual activities at each editable Cs were considered (see the “median” panel, Figure S2a, Supporting Information), the median level by AncBE4max was 15.5%, while the efficiencies by the top three double‐substitution variants reached 37.3% (S55R‐N1317R), 41% (S55R‐A1322R) and 38.9% (N1317R‐A1322R). Interestingly, the introduction of additional substitutions did not appear to have a further positive effect, so that the double‐substitution (2XR) variants generally ranked near the top among all combinatorial X‐to‐R variants. On close examination, the triple‐ and quadruple‐substitution variants (3XR and 4XR) often showed somewhat reduced efficiencies compared with their double‐substitution archetypes (Figure 2c). For instance, for the *ALDOB* site 1 AC_5_ target, the S55R‐A1322R variant showed an editing efficiency of 37% (compared to a control level of 11%). On the other hand, triple‐substitution variants bearing an additional T1314R and N1317R respectively presented 32% and 34% efficiencies, and the quadruple‐substitution variant showed an even lower level at 27% (Figure S2a, Supporting Information). We also extended the analyses for the combinatorial X‐to‐R AncBE4max variants by targeting four other sites (Figure S3a, Supporting Information). The trend of slightly better performances by the 2XR variants over the 3XR and 4XR variants could be again noted (Figure S3a, Supporting Information). As the expression of the base editor could influence its editing efficiency,^[^ ^6^ ^]^ we compared the protein levels of the AncBE4max variants in HEK293T cells (Figure S4, Supporting Information). We found that, while the average level of the 2XR group was close to the control nCas9/AncBE4max, most 3XR variants and the 4XR variant showed comparatively lower expression (4XR at the lowest). When comparisons were made within the 2XR group or the 3XR group, some variants (seemingly associated with T1314R substitution) appeared as low expressors. Although the detailed mechanisms underlying such an abundance pattern are currently unclear, the fact that all Xs‐to‐Rs variants are expressed at levels similar to, or lower than the control AncBE4max level further confirmed their enhanced activities (see Figure 2c; Figure S4, Supporting Information). It could be also extrapolated that the activities of variants with more X‐to‐R substitutions (i.e., 3XR and 4XR) are inadvertently offset by their reduced protein expression.

Since BE‐associated indels arise as a consequence of deamination‐dependent base‐excision,^[^ ^23^ ^]^ the relatively higher levels (the actual rates generally remaining low at all sites) of indels in association with the double X‐to‐R variants also support their higher BE activities (Figures S2b and S3b, Supporting Information). This represents an anticipated tradeoff for development of high‐activity BEs, as previously observed.^[^ ^7^ ^]^ Given the general low levels of indels associated with the AncBE4max variants, the benefits of their enhanced activities conceivably outweigh the indel‐associated risks. Collectively, this series of results suggested that combination of two favorable X‐to‐R substitutions represented an effective approach to further promote base‐editing efficiencies.

We next investigated the performances of different single and combinatorial S55R/T1314R/N1317R/A1322R AncBE4max variants in HeLa cells (Figure 2d,e; Figure S5, Supporting Information). We selected five genomic loci from our earlier HEK293T‐based analyses. The results showed that the overall patterns of relative activities from these single, double, triple, and quadruple X‐to‐R variants in HeLa cells were largely similar to those in HEK293T cells (see Figure 2c). Several double X‐to‐R variants ranked high among all variants (Figure 2e). The results further validated S55R‐N1317R, S55R‐A1322R, and N1317R‐A1322R AncBE4max variants as top‐activity editors, respectively showing 1.9‐, 2.1‐, 2.0‐fold increases in activities over the control levels. Our subsequent experiments would focus on these three double‐substitution variants for further characterizations.

### 2.3 Development of eAncBE4max Variants and Comprehensive Characterization of Their Activities

**Figure 3:**
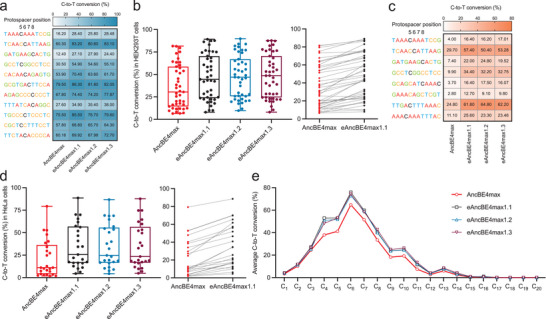
Development of eAncBE4max variants and comprehensive characterization of their activities. a) Heatmap of observed C‐to‐T conversion (%) by AncBE4max and eAncBE4max1.1–1.3 at eleven target contexts in HEK293T cells is presented. Here, we defined S55R‐N1317R, S55R‐A1322R and N1317R‐A1322R nCas9/AncBE4max as eAncBE4max1.1, eAncBE4max1.2 and eAncBE4max1.3, respectively. Black‐colored positions indicate the cytosines of which the editing efficiencies are presented. b) The box plot on the left shows the summary of conversion rates (%) for all C‐to‐T editing positions by AncBE4max and AncBE4max1.1–1.3 in HEK293T cells (from the experiment in (a) and Figure S6, Supporting Information, *n* = 43 editing positions). The center line shows medians of all data points and the box limits correspond to the upper the lower quartiles, while the whiskers extend to the largest and smallest values. The line chart on the right shows a per‐site, paired comparisons between the editing efficiencies of AncBE4max and eAncBE4max1.1. c) The experiment similar to (a) was carried out in HeLa cells at eight target contexts. Heatmap of observed C‐to‐T conversion (%) by AncBE4max and eAncBE4max1.1–1.3 is presented. Black‐colored positions indicate the cytosines of which the editing efficiencies are presented. d) The box plot on the left shows the summary of conversion rates (%) for all C‐to‐T editing positions by AncBE4max and AncBE4max1.1–1.3 in HeLa cells (from the experiment in (c) and Figures S5, S7, Supporting Information, *n* = 25 editing positions). The center line shows medians of all data points and the box limits correspond to the upper the lower quartiles, while the whiskers extend to the largest and smallest values. The line chart on the right shows a per‐site, paired comparisons between the editing efficiencies of AncBE4max and eAncBE4max1.1. e) Average C‐to‐T conversion (%) by AncBE4max and eAncBE4max1.1–1.3 variants at each protospacer position (1 to 20) of the twenty‐three endogenous loci in HEK293T cells (*n* = 23, see Figures 2b,3a; Figures S3a, S6, Supporting Information) are summarized.

After the initial screen for the more active variants of AncBE4max (with engineered nCas9), we named the three top‐activity variants (S55R‐N1317R, S55R‐A1322R and N1317R‐A1322R) as different versions of enhanced AncBE4max (eAncBE4max1.1, eAncBE4max1.2 and eAncBE4max1.3, respectively). To formally benchmark the performances of eAncBE4max1.1–1.3, we selected another eleven sites in HEK293T cells (Figure S6a, Supporting Information). These sites largely feature multiple editable Cs at various positions within the usual editing window (position 4 to 8). The eAncBE4max1.1–1.3 all showed visibly higher levels of editing at a majority of editable positions. To concisely demonstrate the overall effects, the editing levels at representative Cs within the editing windows are presented (**Figure** **3**a). The overall editing efficiencies (medians) from a total of 43 positions by AncBE4max, eAncBE4max1.1, eAncBE4max1.2m and eAncBE4max1.3 in HEK293T cells were 30.5%, 44.5%, 47.0% and 48.6%, respectively (Figure 3b). Furthermore, as mentioned earlier,^[^ ^23^ ^]^ the moderately increased levels (the actual rates generally remaining low at all sites) of indels in association with the eAncBE4max variants are consistent with their higher BE activities (Figure S6b, Supporting Information)

Similar experiments were carried out in HeLa cells for eight additional sites. Likewise, the better performances by eAncBE4max1.1–1.3 over AncBE4max were evident at most editable positions (Figure 3c; Figure S7a, Supporting Information). The indel rates were barely detectable at most sites. At two other sites associated with some editing indels, the levels of such impurities by eAncBE4max1.1–1.3 were correlatively higher, consistent with their patterns of precise editing activities (Figure S7b, Supporting Information). Next, the results on these eight sites and the earlier results on five sites were combined (see Figure S5, Supporting Information). The overall editing efficiencies (medians) from a total of 25 positions by AncBE4max, eAncBE4max1.1, eAncBE4max1.2, and eAncBE4max1.3 in HeLa cells were 11.1%, 25.6%, 24.8%, and 23.5%, respectively (Figure 3d). Therefore, our results in HEK293T and HeLa cells collectively showed that the three versions of eAncBE4max presented largely equivalent levels of activity improvements over AncBE4max.

In results from both cell types, the degrees of improvements by the eAncBE4max variants (version 1.1 as an example) over AncBE4max are mostly consistent for a majority of edited sites (Figure 3b,d, before‐after plots on the right). Interestingly, for a small number of bases that are ranked at the top and bottom in edited percentages (by AncBE4max), the improvement effects by eAncBE4max were diminished. We noted that the bottom‐ranked edits refractory to both AncBE4max and eAncBE4max were always situated outside the main editing window (Figures S6 and S7, Supporting Information). On the other end of the spectrum, it is conceivable that some highly susceptible sites may be edited at “plateau” levels without the need for further editor optimization.^[^ ^7^ ^]^ This could be exemplified by the results on two often investigated sites,^[^ ^24^ ^]^ with abundant Cs (site A and site B, Figure S6a, Supporting Information). Indeed, titrations of the transfected amount of AncBE4max showed that near‐maximal editing within the optimal window of site B (position 6) could be achieved even with AncBE4max transfected at 1/9 of the normal dosage (100 ng) (Figure S8, Supporting Information). In contrast, for several other sites where editing by AncBE4max at C4, C5, or C6 position was less efficient, an editor dose‐correlated effect was observed (between 1/9 and 2/3 of the normal dosage), with an eventual saturation pattern at high dose (see sites from *ALDOB*, *HSP90AB1* and *USP1* in Figure S8, Supporting Information). For such sites, eAncBE4max‐s apparently exhibited greater activities over the control AncBE4max at all doses tested.

We next formally assessed whether eAncBE4max1.1–1.3 altered the original editing windows of AncBE4max. To this end, we combined the results on all 23 target sites from HEK293T cells (Figures S2, S3 and S6, Supporting Information), and averaged the editing efficiencies by the control and eAncBE4max variants at all Cs from position 1 to 20. As expected, AncBE4max induced high levels of editing from C4 to C8, while it also presented lower activities at other positions from C2 to C11 (Figure 3e). Interestingly, eAncBE4max1.1–1.3 only engaged higher editing at Cs between position 4–11, which represent a majority of AncBE4max targets. On the other hand, although AncBE4max could induce certain measurable editing at C2s and C3s, no improvements by eAncBE4max1.1–1.3 were observed at the latter sites. Therefore, compared with the original AncBE4max, the eAncBE4max1.1–1.3 showed no shifts in the editing window (Figure 3e), consistent with their intact overall architecture. Such a feature with eAncBE4max1.1∼1.3 is indeed different from those shown for other previously optimized BEs.^[^ ^7^, ^23^, ^24^, ^25^, ^26^ ^]^

### 2.4 Optimization at the nCas9 Moiety as an Adaptable Strategy to Enhance Various Versions of Cytosine Base Editors, Split‐BE, and Adenine Base Editors

**Figure 4:**
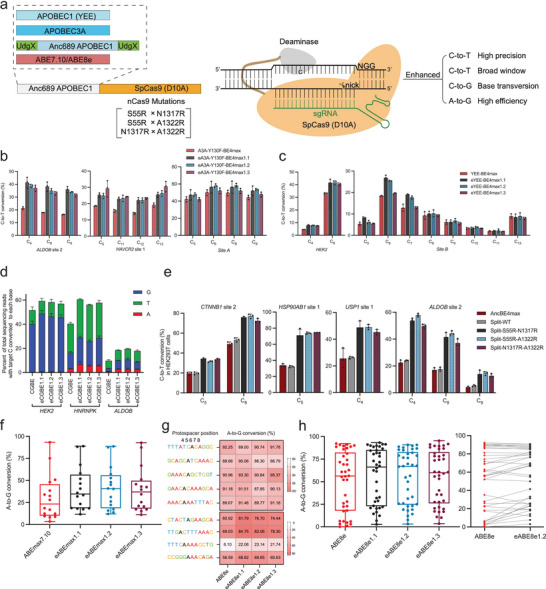
Optimization at the nCas9 moiety as an adaptable strategy to enhance various versions of cytosine base editors, split‐BE, and adenine base editors. a) Schematic diagram of engineered nCas9 variants in combination with other deaminases. b) The A3A‐Y130F‐BE4max was constructed by replacing the Anc689 deaminase with A3A‐Y130F in the AncBE4max backbone. Similar as the nomenclature used earlier, we defined S55R‐N1317R, S55R‐A1322R, and N1317R‐A1322R nCas9/A3A‐Y130F‐BE4max as eA3A‐Y130F‐BE4max1.1–1.3. C‐to‐T conversion (%) at three target contexts in HEK293T cells is presented (means ± s.d., *n* = 3 biological replications). c) We next defined S55R‐N1317R, S55R‐A1322R and N1317R‐A1322R nCas9/YEE‐BE4max as eYEE‐BE4max1.1, eYEE‐BE4max1.2 and eYEE‐BE4max1.3, respectively. C‐to‐T conversion (%) at two target contexts in HEK293T cells is presented (means ± s.d., *n* = 3 biological replications). d) We next introduced X‐to‐R substitutions into CGBE (UdgX‐Anc689‐UdgX‐nCas9‐RBMX). We defined S55R‐N1317R, S55R‐A1322R and N1317R‐A1322R nCas9/CGBE as eCGBE1.1, eCGBE1.2 and eCGBE1.3, respectively (see Experimental Section). The percent of total sequencing reads with the target C respectively converted to T/G/A bases are shown. Data are presented as means ± s.d. (*n* = 3 biological replications). e) C‐to‐T conversion (%) induced by AncBE4max, split‐AncBE4max (split‐WT) and the S55R‐N1317R‐, S55R‐A1322R‐ and N1317R‐A1322R‐introduced split‐BEs at four target contexts in HEK293T cells are shown. Data presented are means ± s.d. (*n* = 3 biological replications). f) We defined S55R‐N1317R‐, S55R‐A1322R‐ and N1317R‐A1322R‐modifed ABEmax7.10 as eABEmax1.1, eABEmax1.2 and eABEmax1.3, respectively. Eight genomic loci were subjected to editing in HEK293T cells. The box plot summarizes the results from all edited adenines (*n* = 16 edited positions, detailed data in Figure S12, Supporting Information). The center line shows medians of all data points and the box limits correspond to the upper the lower quartiles, while the whiskers extend to the largest and smallest values. g) The favorable 2XR‐modifications were similarly introduced into ABE8e to establish eABE8e‐s. Nine genomic loci were subjected to editing by ABE8e and eABE8e‐s in HEK293T cells. Heatmap of observed A‐to‐G conversion (%) by ABE8e and eABE8e1.1–1.3 at different sites is presented. Black‐colored positions indicate the adenines of which the editing efficiencies are presented. h) The box plot on the left summarizes the results from all edited adenines by ABE8e and its variants (*n* = 38 edited positions, detailed data in Figure S13, Supporting Information). The center line shows medians of all data points and the box limits correspond to the upper the lower quartiles, while the whiskers extend to the largest and smallest values. The line chart on the right shows a per‐site, paired comparisons between the editing efficiencies of ABE8e and eABE8e1.2.

The employment of various deaminases in different versions of CBEs leads to their distinct editing characteristics,^[^ ^4^ ^]^ each of which may be more suited for certain application contexts. Given the common nCas9 moiety in diverse base editors, its S55R‐N1317R, S55R‐A1322R, and N1317R‐A1322R variants established above could also contribute to enhancing other CBEs. We next tested such a hypothesis in the models of two additional, well‐characterized CBEs, i.e., A3A‐Y130F‐BE4max and YEE‐BE4max (**Figure** **4**a).^[^ ^27^, ^28^ ^]^ The former was known for its high efficiency and wide editing window, whereas the latter was developed as a high‐precision editor.

Six genomic loci in HEK293T cells were targeted with A3A‐Y130F‐BE4max and its nCas9‐engineered variants (analogously named as eA3A‐Y130F‐BE4max1.1–1.3). Indeed, greater extents of bystander C‐to‐T editing (e.g., C5/11/12/13 for *HAVCR2* site 1) were observed upon A3A‐Y130F‐BE4max applications, with the earlier AncBE4max data as a reference (Figure 4b, and see Figure S6a, Supporting Information for comparison). The results also demonstrated that for the less susceptible sites (i.e., *ALDOB* site 2 and *HAVCR2* site 1), the eA3A‐Y130F‐BE4max1.1∼1.3 groups of editors all showed significantly higher efficiencies than the control (Figure 4b). However, for other four sites where the control A3A‐Y130F‐BE4max exhibited high efficiencies (including site A and site B), all editors presented similar levels of activities regardless of modifications on nCas9 (Figure 4b; Figure S9, Supporting Information). We reason that due to the high potency of A3A‐Y130F‐BE4max, its editing activities at the latter four more susceptible loci might become insensitive to nCas9‐enhanced modifications. Similar “plateau” effect is discussed earlier (see section related to Figure 3b; Figure S8, Supporting Information).

Additionally, the editing efficiencies of the control and the engineered groups of YEE‐BE4max (analogously named as eYEE‐BE4max1.1–1.3) were compared. Two genomic loci were targeted for editing in HEK293T cells. Based on the editing patterns of the C‐abundant “site B”, we first validated that YEE‐BE4max featured an evidently narrowed window where bystander editing besides the top‐edited C6 position was reduced (Figure 4c, and see Figure S6a, Supporting Information for comparison), in line with previous observations.^[^ ^28^ ^]^ Two eYEE‐BE4max variants (i.e., 1.1 and 1.2) showed substantially enhanced activities over the control YEE‐BE4max for the top‐edited cytosines at site B. For another locus (*HEK2*), all three eYEE‐BE4max variants presented higher activities than the control editor (Figure 4c).

Subsequently, we postulated that the engineered nCas9 might also enhance the CGBE that enables C‐to‐G edits following an initial event of nCas9‐programmed cytidine deamination.^[^ ^5^ ^]^ Likewise, nCas9 variants described above were incorporated into CGBE to construct eCGBE1.1∼1.3. Three genomic loci were targeted by the control and eCGBEs in HEK293T cells. These loci underwent low (i.e., *ALDOB* site 1) or notable (i.e., *HEK2* and *HNRNPK* site 1) levels of editing by control CGBE (Figure S10a–c, Supporting Information). For each locus, substantial C‐to‐G edits occurred at the readily‐edited positions (6–10 in a target‐dependent manner). Nevertheless, noticeable levels of C‐to‐T conversions remained at all three loci, along with much lower levels of C‐to‐A conversions detected at two loci. Importantly, compared to control CGBE, the application of eCGBEs resulted in visibly enhanced overall base conversion (C‐to‐G/T/A) levels at all three loci (Figure S10a–c, Supporting Information). The relatively higher rates (the actual rates remaining low at all sites) of indels by eCGBEs are in line with their higher BE activities than CGBE (Figure S10d, Supporting Information).^[^ ^23^ ^]^ The conversion levels of the most edited cytosines at each locus are presented together for a collective view (Figure 4d, with the corresponding, normalized levels highlighted in Figure S10a–c, Supporting Information). The results demonstrated that eCGBEs enabled higher total levels of C‐to‐G conversions than control CGBE at all loci (Figure 4d), while they possibly led to slight increases in ratios of C‐to‐G/(all conversions) (Figure S10a–c, Supporting Information).

Given the attributes of S55R‐N1317R, S55R‐A1322R, and N1317R‐A1322R modifications in nCas9 to generally enhance cytosine base editors, we further considered to apply a similar approach to split‐BE, a popular architecture that circumvents the BEs’ size limitations for key applications such as in vivo editing.^[^ ^29^ ^]^ The split version of control and eAncBE4max‐s were constructed, so that each component respectively harbored a portion of BE and a half Npu intein. When co‐delivered to the cells, the two components can undergo protein trans‐splicing and produce functional BE. We used such split‐BEs to target several sites in HEK293T cells (Figure 4e; Figure S11a, Supporting Information). At these sites, the full‐length and split versions of AncBE4max presented similar editing rates, reminiscent of previous findings.^[^ ^29^ ^]^ Moreover, all split versions of eAncBe4max‐s showed evidently increased levels of editing over the split control BE (Figure 4e; Figure S11a, Supporting Information). The indel rates by these split AncBE4max variants, though at low levels, showed a correlated pattern (Figure S11b,c, Supporting Information). We also determined the levels of all functional components of split AncBE4max and eAncBE4max‐s. The split BEs were tagged at the N‐ and C‐terminus with HA and Flag, respectively. The cells were then transfected with various split BEs as in the editing experiments. WB analyses showed that for all samples of split AncBE4max and eAncBE4max‐s, the levels of the split parts and the full‐length products were comparable (Figure S11d,e, Supporting Information).

Following the validations on various cytosine base editors, we extended our analyses to adenine base editors (ABE). We began with the introduction of S55R‐N1317R, S55R‐A1322R, and N1317R‐A1322R nCas9 modifications to an earlier version of ABE (ABEmax, derived from ABE7.10).^[^ ^6^ ^]^ We found that these favorable 2XR modifications (eABEmax1.1–1.3) could generally enhance the activities of ABEmax in HEK293T cells (Figure 4f; Figure S12, Supporting Information). Subsequently, similar engineering was made on the more recent, high‐activity ABE8e.^[^ ^9^ ^]^ We found that for sites not edited at the maximal rate (<60% conversion at the top‐edited position), an overall improvement of editing could be enabled by the 2XR substitutions (Figure 4g,h; Figure S13, Supporting Information). On the other hand, we found that at sites where the control ABE8e was already highly effective (>80% conversion at the top‐edited position), the 2XR‐modified ABE8e showed limited enhancement effects. These results implicate that the present nCas9 modifications would be suitable to aid those ABE8e applications still challenged by sub‐optimal efficiencies in relevant cells and models.^[^ ^9^ ^]^ Collectively, the results from this series of experiments demonstrated the adaptability of 2XR‐modified nCas9 to enhance a variety of base editors.

### 2.5 The Compatibility of S55R‐N1317R, S55R‐A1322R and N1317R‐A1322R Enhancement Mutations with High‐Fidelity Versions of nCas9 in BE

We next considered the editing fidelities by eAncBE4max‐s. For the assessment of nCas9‐dependent off‐targeting rates, we chose to edit cells with an sgRNA (targeting *FANCF* locus) with well‐characterized off‐target sites.^[^ ^8^ ^]^ We analyzed the levels of control and enhanced AncBE4max‐mediated base editing at the desirable target and seven previously documented nCas9 off‐target sites by NGS. As expected, the eAncBE4max‐s showed higher levels of on‐target base editing compared to the control AncBE4max (Figure S14a, Supporting Information). Indeed, certain nCas9‐dependent off‐target base editing was detected at 4 out of seven sites by all editors, albeit generally at low levels (Figure S14a, Supporting Information). It was interesting to note that eAncBE4max‐s caused moderately higher levels of such off‐target editing, which might represent an undesirable trade‐off for their higher activities.

Spurious, nCas9‐independent base editing by the deaminase domain toward genomic DNA or cellular RNA represents a safety challenge for some base editors.^[^ ^4^, ^30^ ^]^ Nevertheless, it was conceivable that our optimization approach via engineered nCas9 moiety had not instigated such a class of off‐target effects. To verify this notion, we next compared the levels of unintended RNA editing by eAncBE4max with those by AncBE4max via parsing the RNA‐seq data. Consistent with previous observations,^[^ ^31^ ^]^ transfection of AncBE4max resulted in detectable, but low levels of C‐to‐U edits in the transcriptome (Figure S14b, Supporting Information). Of note, the incidence of such Cas‐independent RNA off‐targeting was not exacerbated in eAncBE4max‐transfected cells, consistent with the employment of the same deaminase in the prototype BE and in our engineered editors. Taken together, our results showed that in comparison to the original AncBE4max, its enhanced counterparts (versions 1.1–1.3) did not present further risks of uncontrolled off‐target editing, while they might moderately increase the rates of nCas9‐dependent off‐targeting.

**Figure 5:**
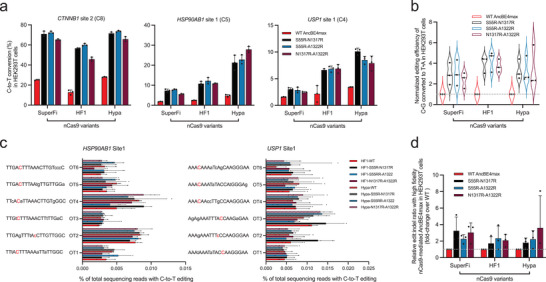
The compatibility of S55R‐N1317R, S55R‐A1322R, and N1317R‐A1322R enhancement mutations with high‐fidelity versions of nCas9 in BE. a) We constructed high‐fidelity versions of BEs by replacing nCas9 in AncBE4max backbone with SuperFi, HF1 and Hypa nCas9,^[^ ^32^ ^]^ respectively. S55R‐N1317R, S55R‐A1322R, and N1317R‐A1322R substitutions were further introduced into these various HF‐BEs. C‐to‐T conversion rates (%) by these variants of SuperFi‐, HF1‐, and Hypa‐nCas9/BEs at three target contexts in HEK293T cells are shown. Data are presented as means ± s.d. (*n* = 3 biological replications). b) Results in (a) were further analyzed by considering editing efficiencies at all sites (*n* = 3 sites) as a whole. The editing frequencies induced by the corresponding HF‐BEs (SuperFi, HF1, and Hypa nCas9) were set as 1, and indicated by the gray dashed line. c) Off‐target analyses for the indicated editing applications (when targeting the *HSP90AB1* site 1 and *USP1* site 1) by HF1‐ and Hypa‐AncBE4max and their enhanced variants in HEK293T cells. Mismatched nucleotides within the off‐target sequences are indicated in lowercase on the left. Data are presented as means ± s.d. (*n* = 3 biological replications). d) Relative edit:indel ratios associated with high‐fidelity versions of BEs (corresponding to data in (a)) are shown. The levels by the HF‐AncBE4max groups were set as 1. Data are presented as means ± s.d. (*n* = 3 biological replicates).

One effective approach to mitigate Cas9‐dependent off‐targeting by BE is via adopting the high‐fidelity (HF) versions of nCas9.^[^ ^20^ ^]^ It is also worth mentioning that most specificity‐improved Cas9 variants paradoxically featured lower activities.^[^ ^18^, ^19^, ^32^ ^]^ Therefore, we speculated that the introduction of the above double X‐to‐R substitutions to a family of HF‐nCas9‐bearing BEs might not only alleviate their sub‐optimal activities, but also minimize the potential nCas9 off‐targeting issues associated with the eBEs. Such combinatorial optimization is mechanistically plausible, as most high‐fidelity Cas9 variants bear modifications of residues (into non‐positively‐charged residues) in domains interacting with the DNA/sgRNA duplex,^[^ ^22^, ^33^, ^34^, ^35^ ^]^ or in the HNH domain,^[^ ^18^ ^]^ apparently independent of the S55/N1317/A1322 motif (in a region neighboring the PAM‐proximal NTS) (see Figure 1a). To this end, we respectively placed S55R‐N1317R, S55R‐A1322R and N1317R‐A1322R modifications into three previously established HF‐nCas9 variants, i.e., SuperFi‐, HF1‐ and Hypa‐nCas9,^[^ ^22^, ^33^, ^35^ ^]^ within the AncBE4max architecture. In the ensuing experiments, these new base editors were each used to edit three loci tested earlier in HEK293T cells (**Figure** **5**a,b and see Figure 2b for the editing rates of AncBE4max). In reference to the original AncBE4max, its HF‐nCas9‐bearing counterparts all showed markedly reduced base‐editing activities. On the other hand, the HF‐nCas9/AncBE4max‐s were indeed associated with undetectable levels of off‐target editing (Figure 5c). The results also showed that the introduction of S55R‐N1317R, S55R‐A1322R or N1317R‐A1322R modifications to HF‐nCas9‐bearing AncBE4max‐s led to notable alleviation of the latter's low on‐target BE activities (Figure 5a,b). Importantly, such modifications did not compromise the superior on‐target specificity featured by the HF‐nCas9 BEs (Figure 5c). The application of HF‐nCas9 BEs was however associated with low levels of on‐target indels. For 2 (out of 3) HF‐nCas9 BEs, the double X‐to‐R variants showed some elevation in indel levels (Figure S15, Supporting Information). Nevertheless, the latter modifications on HF‐nCas9 BEs consistently improved their edit: indel ratios (Figure 5d). These series of results validated that merging our enhancement nCas9/BE modification into HF nCas9/BE could further optimize high‐safety base editing to achieve much improved efficiencies.

### 2.6 Mechanistic Dissection of BE Enhancement by the Use of Engineered Cas9 Variants

To directly examine the activities of the engineered Cas9 variants, we introduced S55R‐N1317R, S55R‐A1322R and N1317R‐A1322R substitutions into cleavage‐competent Cas9. The activities of these Cas9 variants were tested on six genomic target sites where eAncBE4max‐s had exhibited higher efficiencies over AncBE4max (Figure S2 and S3, Supporting Information). While the WT Cas9 potently induced indels at these loci (from 63% to 93%), all three engineered Cas9 variants exhibited somewhat higher activities at five out of six sites (Figure S16a,b, Supporting Information). The only exception was at *PRNP* site 1, where the WT Cas9 already enabled the highest (93%) indel rate among all sites tested. Therefore, the activities of engineered Cas9 and their eAncBE4max counterparts correlated modestly. Through another series of experiments, we verified that the S55R‐N1317R, S55R‐A1322R, and N1317R‐A1322R modifications of various HF versions of Cas9 nuclease markedly alleviated their typical low indel‐forming activities (Figure S16c,d, Supporting Information), in general agreement with the earlier base‐editing results (see Figure 5a,b). Therefore, besides base editing, our enhancement strategy may also apply to Cas9 editing, particularly in the context of using HF‐Cas9 forms.

**Figure 6:**
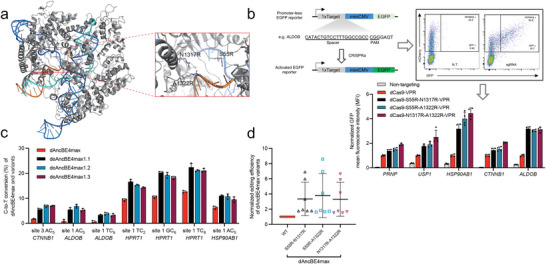
Mechanistic dissection of BE enhancement by the use of engineered Cas9 variants. a) To simulate the side‐chain positioning of the engineered arginine residues (R55, R1317, and R1322), we respectively introduced the according substitutions into the structural model of Cas9/sgRNA/DNA complex (PDB: G81I). The zoomed in portion on the right shows that the side chains of R55 and R1322 are positioned on either side of the characteristic DNA kink at the initial segment of the unwound NTS. b) The abilities by dCas9‐modified (S55R‐N1317R, S55R‐A1322R, and N1317R‐A1322R) CRISPRa devices to drive targeted activation of the EGFP reporters were compared. The activities were tested respectively with five sgRNA and their corresponding reporters, similar to a previous work.^[^ ^53^ ^]^ The top part shows the assay scheme, as well as a representative flow cytometry result indicative of CRISPRa activity in HEK293T cells (using dCas9‐VPR‐S55R‐A1322R editor and sgRNA targeting the site in the *ALDOB* gene; N.T. refers to a non‐targeting sgRNA). On the bottom, the graph displays the summarized results on the control‐ and dCas9‐modified CRISPRa activities. The Mean Fluorescence Intensity (MFI) values from the EGFP reporter were normalized by those from mCherry. The normalized values indicate CRISPRa activities (mean ± s.d., *n* = 4 biological replicates). c) C‐to‐T conversion (%) induced by the dCas9‐based, d‐AncBE4max, and d‐eAncBE4max1.1–1.3 at four target contexts (total of seven editing positions) in HEK293T cells are shown. Data presented are means ± s.d. (*n* = 3 biological replications). d) Results in (c) is further analyzed by considering all editing events (*n* = 7 editing positions) as a whole. The editing frequencies induced by dAncBE4max were set as 1.

To simulate the side‐chain positioning of the engineered arginine residues (R55, R1317, and R1322), we respectively introduced the according substitutions into the structural model of Cas9/sgRNA/DNA complex (PDB: 8G1I) using PyMOL software (**Figure** **6**a). It was clear from such simulation that the side chains of R1322 and R55 were positioned bilaterally near the characteristic, kinked DNA at the initial part of the unwound NTS. It is therefore conceivable that the positive charges on R55 and R1322, respectively, reinforce the interactions of the S55R‐N1317R, S55R‐A1322R, and N1317R‐A1322R Cas9 variants with the DNA target. On the other hand, as the side chain of R1317 appears further away from the NTS DNA in comparison to that of R1322, the N1317R substitution (and potentially S55R and A1322R) may also engage other enhancement mechanisms.

To experimentally validate the improved interactions between S55R‐N1317R, S55R‐A1322R, and N1317R‐A1322R nCas9 moiety and the DNA target, we employed the CRISPRa assay (dCas9‐VPR) as a proxy to assess Cas9‐DNA binding.^[^ ^36^ ^]^ Given the mutations at the active sites (D10A‐H840A) of both RuvC and HNH nuclease domains, the activities of the engineered dCas9‐VPR variants would reflect their target binding abilities. The enhancement 2XR substitutions were hence introduced into dCas9‐VPR. Additionally, customized reporter plasmids were constructed by directly inserting specific genomic target sequences upstream of a promoter‐less (miniCMV) EGFP cassette (Figure 6b). As a result, the activities of dCas9‐VPR variants would be measured by their induction of the EGFP fluorescent reporter. The dCas9‐VPR variants were respectively transfected together with each set of sgRNA and the corresponding reporter into HEK293T cells. The 2XR modifications of dCas9‐VPR led to significant increases of CRISPRa activities (Figure 6b), demonstrating their positive impacts on dCas9‐target binding.

Given their more stable target binding, the engineered nCas9 (S55R‐N1317R, S55R‐A1322R, and N1317R‐A1322R) moieties could enhance base editing via two intermediate effects: 1) to enable more efficient cytosine deamination by the anchored APOBEC enzyme, and 2) to subsequently potentiate target strand nicking, as to facilitate the eventual installation of edits. To further dissect these possibilities, we considered examining the base editing activities of the dCas9 version of eAncBE4max‐s at four genomic loci investigated earlier. Compared to the usual nCas9‐based system (see Figure S2 and S3, Supporting Information), such nick‐independent base editors exhibited suboptimal activities (Figure 6c), which highlighted the benefit of target strand nicking to the installation of edits.^[^ ^1^ ^]^ Importantly, similar to the eAncBE4max1.1–1.3, their dCas9‐equiped counterparts presented substantially higher activities over the control dCas9/AncBE4max (Figure 6c,d). Indeed, the correlations between the results with the nCas9‐ or dCas9‐based AncBE4max variants were strong, as the respective *R* ^2^ values for different sites were from 0.86 to 0.96 (Figure S17, Supporting Information). These results are consistent with a model that the improvement of target binding by the engineered nCas9 (S55R‐N1317R, S55R‐A1322R, and N1317R‐A1322R) contributes directly to the more efficient base editing by our enhanced base editors.

### 2.7 Application of Enhanced BE in Primary Human *T*‐Cell Editing

**Figure 7:**
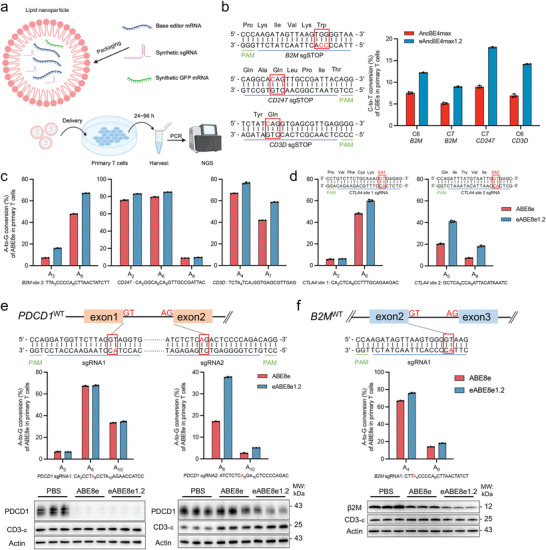
Application of enhanced BEs in primary human *T*‐cell editing. a) Schematic diagram of LNP‐mediated delivery of BE mRNA and synthetic sgRNA for editing primary human *T*‐cells. b) On the left, sgRNA‐targeted sequences in *B2M*, *CD247*, and *CD3* are displayed. The substrate cytosines whose conversion would lead to a stop codon are marked in red color, while the positions corresponding to the stop codon are boxed. Four days following the delivery of CBE mRNA (AncBE4max or eAncBE4max1.2)/sgRNA into the in vitro‐activated primary human *T*‐cells, the cells were harvested for targeted NGS analyses. On the right, the base conversion rates at the substrate cytosines are graphed. Data are presented as means ± s.d. (*n* = 3 biological replications). c) For parallel assessments, the same CBE target sites (in *B2M*, *CD247*, and *CD3D*, as in (b)) were also subjected to editing by ABE8e and eABE8e1.2. A‐to‐G conversion rates (%) are shown. Data are presented as means ± s.d. (*n* = 3 biological replications). d) ABE8e and eABE8e1.2 were adopted to target the splicing junction sites in *CTLA4* gene (*CTLA4* site 1and *CTLA4* site 2). The targeted splice donors are highlighted by red boxes. A‐to‐G conversion rates (%) are shown. Data are presented as means ± s.d. (*n* = 3 biological replications). e and f) The top diagrams illustrate the use of ABE8e and eABE8e1.2 to target a pair of splicing donors and acceptors in the *PDCD1* gene e), or a splicing donor in the *B2M* gene f). The corresponding A‐to‐G conversion rates (%) are shown below the splicing junction diagrams. Data are presented as means ± s.d. (*n* = 3 biological replications). Accordingly, the Western blot analyses for PD‐1 e) and β2 M protein f) are also presented.

BE induces programmable mutations independent of DSB generations, and therefore represents a potentially safer alternative to Cas9 nuclease.^[^ ^4^ ^]^ Currently, there is a strong interest from the field of cancer immunotherapy, in the development of “universal” CAR‐T cells that require inactivation of genes driving graft‐versus‐host disease, graft rejection, and/or *T*‐cell‐checkpoint responses.^[^ ^37^ ^]^ Therefore, to further establish the application potential of our eBE platform, we adopted the control and engineered BEs to edit relevant targets in human *T*‐cells (**Figure** **7**a).

CBE could induce premature stop codons for gene inactivation.^[^ ^38^ ^]^ We first selected several popular targets for human *T*‐cells, i.e., *B2M*, *CD247*, and *CD3D*, and designed corresponding sgRNAs for the introduction of non‐sense mutations (Figure 7b). The LNP vector was used for the delivery of CBE mRNA (AncBE4max or eAncBE4max1.2)/sgRNA into the in vitro‐activated primary human *T*‐cells.^[^ ^39^, ^40^ ^]^ Four days after delivery, the cells were harvested. The programmed base conversion rates were determined by targeted NGS analyses. Although the overall C‐to‐T editing efficiencies were moderate (5–20%), it was apparent that eAncBE4max1.2 induced significantly higher rates of base conversions than AncBE4max (Figure 7b). Compared with published standards of CBE efficiencies in T cells,^[^ ^38^, ^41^ ^]^ we envision that further optimization of cultural conditions, editor formats, and/or delivery protocols may formally unleash the application potential of our eCBE‐s in editing primary T cells.

On the other hand, the A‐to‐G editor, particularly the high‐activity ABE8e could serve as an alternative tool for human *T*‐cell editing.^[^ ^41^ ^]^ Given our establishment of eABE8e variants (Figure 4f–h; Figure S12 and S13, Supporting Information), we prepared LNP‐encapsulated ABE8e or an enhanced variant (eABE8e‐1.2) to edit human *T*‐cells. For parallel assessments, the same CBE target sites (in *B2M*, *CD247*, and *CD3D*) were subjected to editing by the ABE8e‐s. Indeed, the results from these *T*‐cell experiments showed that compared to AncBE4max‐s, ABE8e‐s exhibited much greater base conversions rates (48∼85% A‐to‐G at the CBE sites] (Figure 7c, see Figure. 7b for reference). Furthermore, eABE8e‐1.2 also presented a noticeable increase in activities over ABE8e (Figure 7c).

ABE could be used to target splicing junctions to also drive gene inactivation. Toward this end, we further applied LNP‐ABE8e and ‐eABE8e‐1.2 to target the splicing junctions in several relevant genes, i.e., *CTLA4*, *PDCD1*, and *B2M* (Figure 7d–f). The NSG analyses showed that at 4 out of 5 targeted sites, eABE8e‐1.2 induced higher levels of base conversions than the control ABE8e (quantitation in Figure 7d–f). The one exception was for editing a splicing donor in *PDCD1* (Figure 7e left), where both ABE8e and eABE8e‐1.2 enabled ≈65% A‐to‐G conversions, causing similar degrees PD‐1 protein ablation. On the other hand, consistent with the overall higher base‐editing activity of eABE8e‐1.2 (than ABE8e) at other splicing junctions, more effective knock‐down of the corresponding proteins was observed in other eABE8e samples (Figure 7e right and 7f). Overall, these results illustrate the superior activity of our eABE8e over the control ABE8e in human *T*‐cells (Figure S18, Supporting Information), and implicate the broad potential of the presently engineered BE variants in the development of advanced *T*‐cell‐based therapies.

## 3 Discussion

CRISPR/Cas9‐derived base editors harness various cytidine and adenosine deaminase domains to enable targeted base conversions, without requiring to generate DSB and to co‐deliver the repair template.^[^ ^1^, ^2^ ^]^ Since their introduction to the genome‐editing field, the BEs have been rapidly adopted for the generation of disease models,^[^ ^42^ ^]^ plant engineering,^[^ ^43^ ^]^ and development of gene/cell therapy,^[^ ^44^, ^45^, ^46^, ^47^ ^]^ showing significant promise in future application. Many recent developments for improvement of the BE tools have been centered on expanding the choices of cytidine and adenosine deaminase domains, and to optimize their activity/safety profiles.^[^ ^4^ ^]^ Despite these efforts, the BE efficiencies still present challenges, especially for applications in therapeutic models.^[^ ^9^, ^48^ ^]^ Therefore, the present study explored nCas9‐engineering as a potentially general strategy to enhance various versions of base editors.

Through first testing a number of point mutants of nCas9 in the architecture of AncBE4max, we subsequently came to adopt an enhancement strategy of engineering combinatorial Xs‐to‐Rs substitutions in a region of nCas9 neighboring the PAM‐proximal NTS (Figures 1, 2 and 3).^[^ ^3^, ^12^, ^13^ ^]^ The top‐performing modifications of S55R‐N1317R, S55R‐A1322R, and N1317R‐A1322R drove consistent enhancement of actual editing rates by AncBE4max (eAncBE4max1.1–1.3), so that the typically less susceptible targets can often be edited at ≥2‐fold higher levels. Importantly, such engineered nCas9 variants could serve as general modules to enhance various forms of cytosine base editors, and also adenine base editors (Figure 4). It is worth pointing out that for the much‐enthused prospect of in vivo BE, splitting the editor is likely to be required to accommodate its packaging in the safety‐compliant AAV vectors.^[^ ^29^ ^]^ In this regard, the fact that S55R‐N1317R, S55R‐A1322R, and N1317R‐A1322R modifications in split‐AncBE4max constructs also substantially enhanced base editing supports the future development of our new tools toward AAV vector‐mediated application.

A convenient feature of our nCas9‐oriented approach for BE activity enhancement is the less likelihood of inadvertently exacerbating the non‐specific, deaminase‐dependent base editing, which represents a significant safety concern for the development of cytosine base editors.^[^ ^28^ ^]^ Indeed, equivalent non‐specific RNA editing events were observed for the control and enhanced AncBE4max‐s (Figure S14b, Supporting Information). On the other hand, the use of eAncBE4max‐s was associated with moderately greater rates of off‐targeting than the control editor at those nCas9‐dependent sites (Figure S14a, Supporting Information). Notably, our results showed that such a CRISPR/Cas‐related safety caveat of eAncBE4max could be addressed by instead introducing the relevant Xs‐to‐Rs modifications into various forms of high‐fidelity nCas9/AncBE4max. This approach also led to noticeable alleviations of the typical low activities by the HF nCas9/AncBE4max‐s (Figure 5). The spatial separation of the S55/N1317/A1322 motif from most specificity‐determinant regions has readily allowed concomitant optimization of nCas9/BE's fidelity and activity. Although not directly tested in the present study, our enhanced HF‐nCas9/BE platforms are also poised to integrate with a high‐specificity editor architecture or activity‐restrained deaminase domain(s),^[^ ^4^ ^]^ to enable efficient base editing with further optimized safety. Moreover, the present 2XR modifications may be introduced into the PAM‐relaxed nCas9/BEs,^[^ ^49^ ^]^ to empower such high‐flexibility BEs with greater efficiencies.

Given the potential of *T*‐cell genome editing in the development of advanced treatments for cancer,^[^ ^50^ ^]^ we further assessed the performances of the engineered BE platform in human *T*‐cells (Figure 7). The results validated apparently enhanced activities of the engineered AncBE4max and ABE8e over their controls (≈82% and ≈25% increases at median levels, respectively) in human T cells. In these experiments, the group‐best eABE8e‐1.2 achieved a median of ∼60% base conversions at the most edited base positions (Figure S18, Supporting Information), an efficiency approaching the level suited for practical use. Although other aspects of optimizations are also warranted, the data from the *T*‐cell experiments strongly support the application potential of our engineered BEs.

We found that while the corresponding engineering on WT Cas9 could somewhat enhance its cleavage activity, rather prominent improvements could be achieved on the high‐fidelity forms of Cas9 with typically lower efficiencies (Figure S16, Supporting Information). Our strategy also increased the activation potencies of the dCas9‐VPR tool (Figure 6). Together with the fact that similar effects were observed for dCas9‐BE, these data validate a mechanistic model that the improved affinity of the engineered Cas9 moiety with the DNA target underlies the greater activities by eBEs (Figure 6). Furthermore, the results on Cas9 and on dCas9‐VPR strongly suggest the application potential for the presently established Xs‐to‐Rs modifications in other forms of genome editing. Interestingly, only very few enhancement variants based on WT SpCas9 have been previously established through cleavage‐dependent assays,^[^ ^51^, ^52^ ^]^ which may be due to the readily high nuclease activity of WT SpCas9. In this regard, our current explorations initially on the less potent cytosine BE activities may have been more conducive to identifying the enhancement variants of SpCas9. We speculate that more extensive screens of nCas9/BE modifications might represent a productive direction to broadly advance the development of many Cas9‐dependent tools.

## 4 Experimental Section

### Plasmid Construction

AncBE4max, YEE‐BE4max, CGBE (UdgX‐Anc689‐UdgX‐nCas9‐RBMX) plasmids were purchased from Addgene (Addgene, #112094, #138157 and #163552, respectively). For expression of A3A‐Y130F‐BE4max, the plasmid backbone was amplified from P‐CMV‐AncBE4max (Addgene, #112094), then replaced the Anc689 APOBEC with A3A‐Y130F using recombinase‐based cloning (Vazyme, ClonExpress II One Step Cloning Kit, #C112‐02‐AB). When constructing CBEs, CGBE, dCas9‐CBEs, Cas9, nCas9, and dCas9‐VPR mediated by engineered Cas9 variants, circular PCR using specific primers containing the desired variants and specific plasmids containing WT Cas9 as templates, respectively was performed. The sequences information for circular PCR are provided in Table S1 (Supporting Information). Then the PCR product was transformed into E. coli and screened using Ampicillin. For the expression of sgRNA, the plasmid backbone pGL3‐U6‐sgRNA‐mCherry from our previous study was used.^[^ ^53^ ^]^ Then, the plasmid backbone was cut by BsaI‐HFv2 (NEB) for overhangs. The sgRNA spacer oligos (featuring compatible overhangs, top strand with ends of 5′ACCG, bottom strand with 5′AAAC overhang) were synthesized and annealed. Finally, two fragments (annealed spacer and the cut backbone) were assembled by DNA ligase. The sequences information for sgRNAs are provided in Table S2 (Supporting Information). Assembled plasmids were transformed into E. coli and screened using Ampicillin.

### Cell Culture, Transfection, and Harvest

HEK293T (ATCC CRL‐3216) and HeLa (ATCC CCL‐2) cells were cultured in Dulbecco's Modified Eagle Medium (Gibco) supplemented with 10% fetal bovine serum (FBS) (v/v) from Gemini. The cells were incubated at 37 °C with 5% CO2. For plasmid transfection, HEK293T or HeLa cells were seeded on poly‐D‐lysine‐coated 24‐well plates and transfected when they reached ≈70% confluence using EZ Trans (Shanghai Life iLab Biotech Co., Ltd) following the manufacturer's protocols. For base editing, 900 ng pCMV‐CBEs/CGBE/ABEs (for split‐BE, 450 ng split‐BE‐N, and 450 ng split‐BE‐C) plasmid, along with 300 ng sgRNA plasmid (containing an hPGK‐mCherry marker) were transfected into cells per well. For the Cas9 cleavage efficiency assay, 900 ng pCMV‐Cas9 plasmid, along with 300 ng sgRNA plasmid, were transfected into cells per well. After 72 h of transfection, harvest mCherry+ cells by Fluorescence Activated Cell Sorting (FACS) for sequencing analyses.

### Targeted NGS

To analyze the target sites editing efficiency, genomic DNA was extracted and used as a template for PCR amplification, employing the Phanta Max Super‐Fidelity DNA Polymerase (Vazyme). Primers used for HEK293T and HeLa cells are listed in Table S3 (Supporting Information). PCR products with distinct barcodes were mixed for deep sequencing using the Illumina HiSeq X Ten platform (2 × 150 PE) by Annoroad Gene Technology (Beijing, China). Experimental conditions were differentiated by the use of barcodes, and experimental repetitions were grouped accordingly. The sequencing reads were demultiplexed using AdapterRemoval (version 2.2.2), and pair‐end reads with alignments of 11 bp or more were merged into a single consensus read. Subsequently, all processed reads were mapped to the target sequences using the BWA‐MEM algorithm (BWA v0.7.16). To evaluate base editing efficiency, the percentage of reads with the desired edit that does not contain indels was calculated in relation to the total mapped reads. The indel frequency was determined by dividing the number of reads containing indels by the total mapped reads. Lastly, the mutation rate was calculated using bam‐readcount, applying parameters ‐q 20 ‐b 30 ‐i.

### Off‐Target Analysis

Potential off‐target sites were predicted in the human genome (GRCh38/hg38) with Cas‐OFFinder (2.4) (http://www.rgenome.net/cas‐offinder). The sequences around the predicted off‐target sites were amplified using Phanta Max Super‐Fidelity DNA Polymerase (Vazyme), and subjected to NGS with the Illumina HiSeq X Ten (2 × 150 PE) at Annoroad Gene Technology, Beijing, China. The amplicons were analyzed with as the method described in Targeted NGS.

### RNA Off‐Target Analysis by RNA‐Seq

For RNA‐Seq, 1000000 transfected cells (GFP+ for mock transfection groups and mCherry+ for CBEs groups) were sorted by FACS (Moflo Astrios EQ). The TRI‐ ZOL reagent (Vazyme) was used to extract total RNA. The RNA‐Seq libraries were prepared according to standard protocols for the NovaSeq platform and subjected to commercial RNA‐Seq services (Anoroad Genome Institute). The libraries were sequenced at a depth of ≈20 million reads per sample. The reads were mapped to the human reference genome (hg38) by STAR software (Version 2.5.1); annotation from GENCODE version V30 was used. After removing duplication, variants were identified by GATK (Version 4.1.8.1). For MuTect2 method, variants were filtered with FilterMutectCalls. The depth for a given edit should be at least 10x and these edits were required to have at least 99% of reads supporting the reference allele in the wild‐type samples.

### LNP Encapsulation

The CBEs and ABE8e‐s mRNA were in vitro transcribed from T7 promoter‐led templates using the mMESSAGE mMACHINE T7 kit (Invitrogen) and Poly(A) Tailing Kit (Invitrogen). The sgRNA and GFP mRNA (as positive CTRL to indicate LNP delivery) used were synthesized from GenScript (Nanjing, China). Spacer sequences of sgRNA are listed in Tables S4. LNP were prepared by rapid mixing of organic solution and aqueous solution (volume ratio 1:3). The organic solvent mixture consisted of 41.5 mol% SM‐102, 37.5 mol% cholesterol,10 mol% DOTAP, 10 mol% DSPC and 1.5 mol% DMG‐PEG 2000. The LNP encapsulation was accomplished by mixing the lipid constituents (2 mg mL^−1^) suspended in ethanol with mRNA (including BE mRNA, targeting sgRNA and GFP, total 10 pg/cell dose, at mRNA:sgRNA = 1:1 weight ratio, GFP = 250 ng/well) diluted with 50 µL citric acid buffer (0.05 mol L^−1^, pH = 4). The cargo‐encapsulated LNP was immediately diluted with PBS. The buffer was exchanged via ultrafiltration‐based concentration (Amicon Ultra, 100 kDa) to remove ethanol. The final stock was in a total volume of 100 uL, and stored at 4 °C for subsequent utilization.

### T Cell Isolation, Culture and Transfection

Naive CD3^+^ *T*‐cells were isolated from healthy donors following informed consent procedures. The procedure was approved by the Ethics Committee of the Affiliated Drum Tower Hospital of Nanjing University Medical School (Approval #: 2016–027). First, peripheral blood mononuclear cells (PBMCs) were isolated from human whole blood by density gradient centrifugation. Subsequently, CD3^+^ T lymphocytes were further purified by magnetic negative selection using the EasySep Magnetic Cell Isolation Kit (STEMCELL). Then, CD3^+^ *T*‐cells were plated into 48‐well plates at a density of 2.4 × 10^5^ cells per well. CD3^+^ T cells were cultured in RPMI1640 medium. Human T activator CD3/CD28 Dynabeads (head cell ratio 1:1), 2‐mercaptoethanol (50 µm), IL‐2 (300 U mL^−1^), IL‐15 (5 ng mL^−1^) and IL‐7 (5 ng mL^−1^) were added to the culture medium. After CD3^+^ *T*‐cells were activated for 24 h, the cells medium was refreshed with serum‐free TexMACS Medium (Miltenyi) (plus additional IL‐2 (300 U mL^−1^), IL‐15 (5 ng mL^−1^), and IL‐7 (5 ng mL^−1^)), and BE‐encapsulated LNP was added to the cells. 96 h after infection, cells were collected directly for NGS analysis or WB analysis. The primers used are listed in Table S5 (Supporting Information).

### Western Blotting

For Western blotting, 24‐well plate HEK293T cells or 48‐well plate primary *T*‐cells were lysed by RIPA. The primary antibodies used included anti‐Cas9 (Genscript (A01935, clone 4A1), 1:500), anti‐Actin (Abcam (ab124964), 1:1000), anti‐β2M (CST (12851), 1:1000), anti‐PDCD1 (CST (84651), 1:1000), anti‐CD3ε ((CST (4443), 1:1000), anti‐HA‐Tag (CST (3724), 1:1000) and anti‐FLAG M2 (CST (14793), 1:1000). Images were captured with Amersham Imager 600. Uncropped blots for the presented results are provided in the Source Data file.

### CRISPRa Assay Toward the Reporter

In total, HEK293T cells were seeded on poly‐D‐lysine‐coated 24‐well plates and transfected when they reached approximately 60–90% confluency. The transfection involved 3.6 µL of EZ Trans, CMV‐dCas9‐VPR (900 ng), targeted EGFP reporters (30 ng), and various sgRNAs (100 ng). The sgRNA plasmids contained a mCherry marker, which served as a control for transfection efficiency. After a 2‐day transfection, the cells were analyzed using flow cytometry. The EGFP mean fluorescence intensity (MFI) was normalized to that of mCherry, allowing the representation of corresponding CRISPRa activities.

### Statistical Analyses

All the data presented in this study were based on three biological replicates to ensure robustness and reliability. Graphing and statistical analyses were conducted using GraphPad Prism (version 9). The results are presented as means ± s.d., as indicated in the figure legends. Box plots were utilized to represent the data, where the center line corresponds to the median, and the box limits represent the upper and lower quartiles.

## Conflict of Interest

The authors declare no conflict of interest.

## Author Contributions

G.Z., Z.S., S.H., and Y.W. contributed equally to this work. X.H., F.L., X.W., and J.L. conceived the study and designed experiments. G.Z., S.Z., Y.W., and J.S. performed the experiments with the assistance of L.Q., G.L., Y.W., Y.F., W.H., J.T., Y.C., and colleagues of Molecular and Cell Biology Core Facility (MCBCF) at the School of Life Science and Technology of ShanghaiTech University. S.H. analyzed the data. All authors discussed the results and approved the manuscript. G.Z., S.Z., F.L., X.W., and J.L. wrote and revised the manuscript.

## Supporting information

Supporting Information

## Data Availability

The data that support the findings of this study are openly available in National Center for Biotechnology Information Sequence Read Archive database at https://www.ncbi.nlm.nih.gov/bioproject/?term=PRJNA1107383, reference number 1107383.
