# Supplementary material for: nCas9 Engineering for Improved Target Interaction Presents an Effective Strategy to Enhance Base Editing
Source: Adv Sci (Weinh). 2024 Jun 17;11(31):2405426. doi: 10.1002/advs.202405426 (PMC11336945; doi:10.1002/advs.202405426)
Supplement: Supplementary file 1 — Supporting Information [file ADVS-11-2405426-s001.pdf]

ADVANCED SCIENCE
Open Access

Supporting Information

for *Adv. Sci.*, DOI 10.1002/advs.202405426

nCas9 Engineering for Improved Target Interaction Presents an Effective Strategy to Enhance Base Editing

Guiquan Zhang, Ziguo Song, Shisheng Huang, Yafeng Wang, Jiayuan Sun, Lu Qiao, Guanglei Li, Yuanyuan Feng, Wei Han, Jin Tang, Yulin Chen, Xingxu Huang, Furui Liu, Xiaolong Wang* and Jianghuai Liu**

Supporting Information for

nCas9 Engineering for Improved Target Interaction Presents an Effective Strategy to Enhance Base Editing

Guiquan Zhang, Ziguo Song, Shisheng Huang, Yafeng Wang, Jiayuan Sun, Lu Qiao, Guanglei Li, Yuanyuan Feng, Wei Han, Jin Tang, Yulin Chen, Xingxu Huang, Furui Liu, Xiaolong Wang*, Jianghuai Liu**

This PDF file includes.

Figure S1 to Figure S18

Table S1 to Table S5

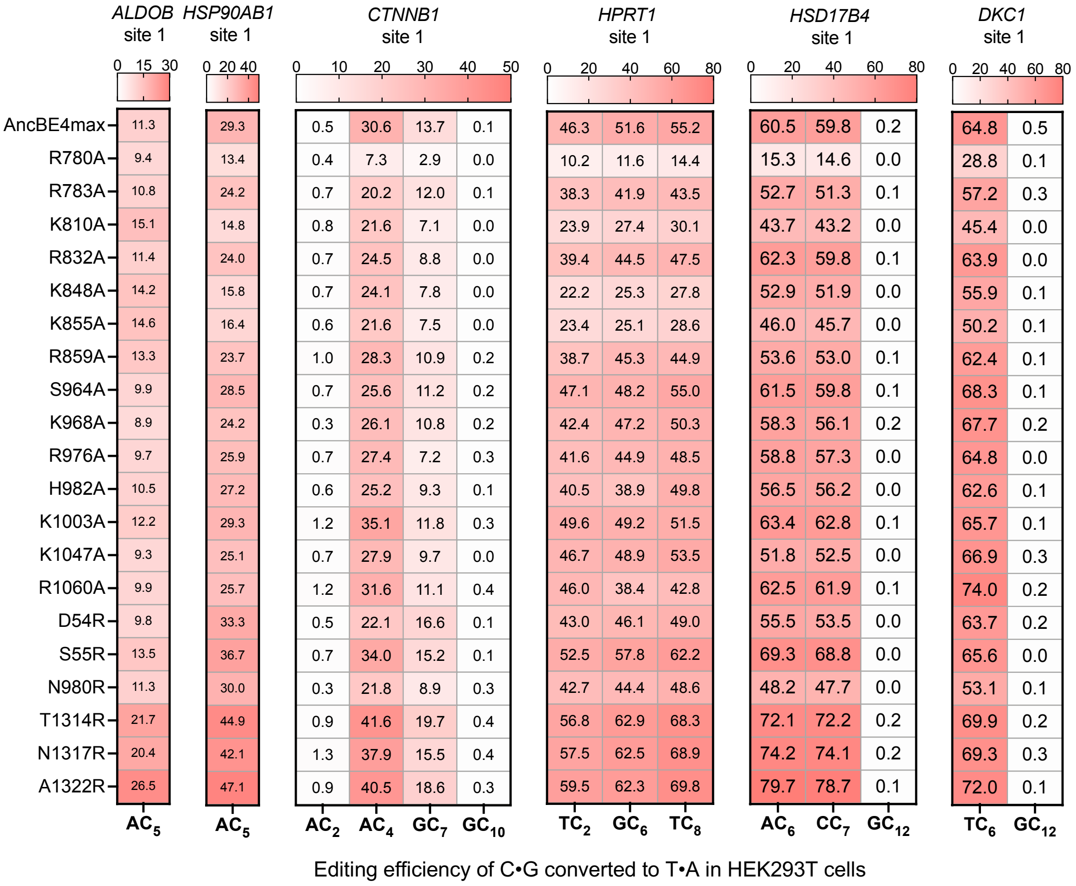

Figure S1. Engineered nCas9 variants improve the editing efficiency of AncBE4max in HEK293T cells. Heatmap of observed C-to-T conversion (%) induced by AncBE4max and 20 engineered AncBE4max variants containing different single mutations in nCas9 at six endogenous genomic loci in HEK293T cells. The results show the editing efficiency of all-edited C-to-T within the editing window, data are presented as means (n = 3 biological replicates).

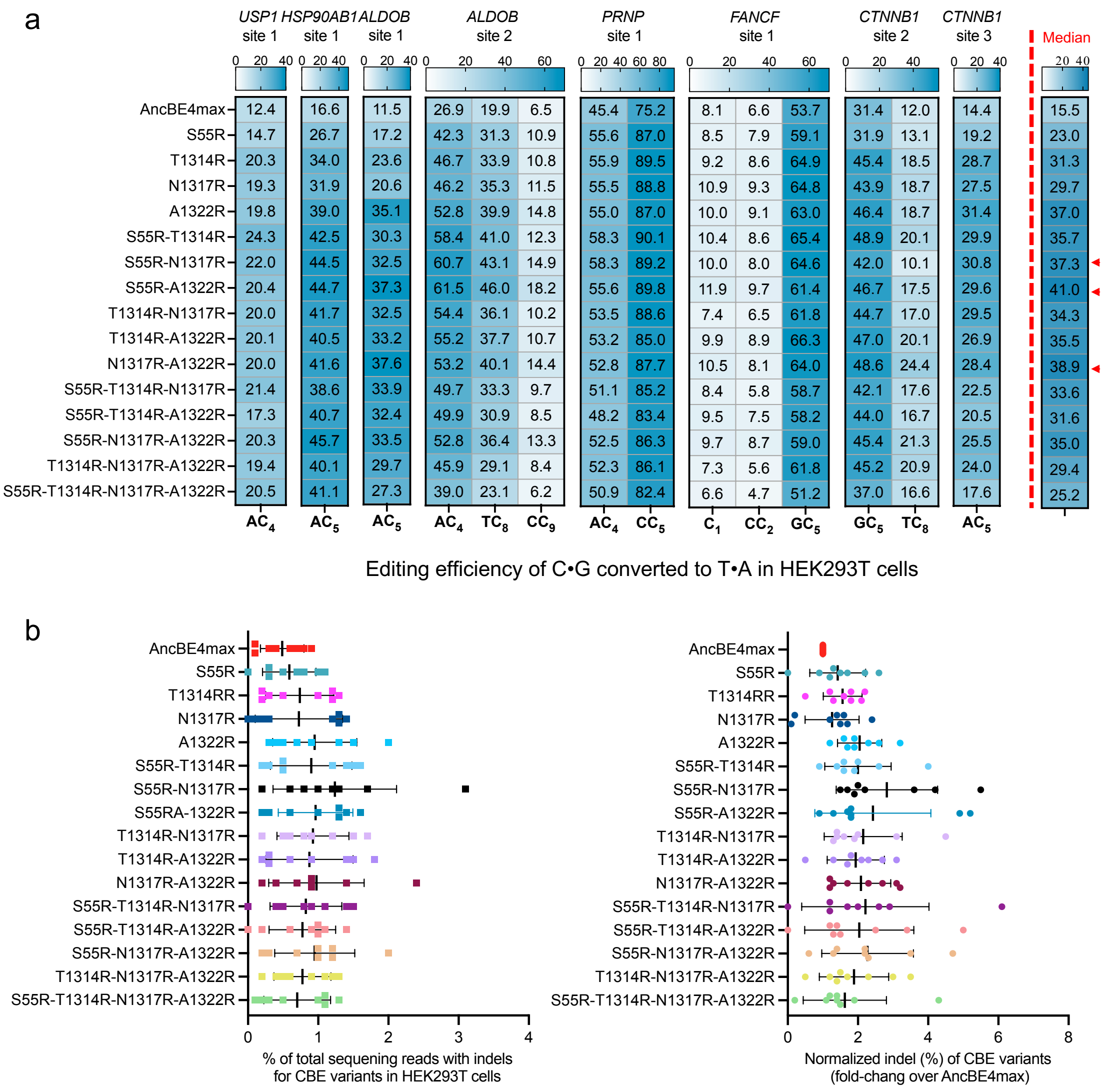

Figure S2. Combination of X-to-R mutations in nCas9 for further improvement of AncBE4max activities at more tested sites in HK293T cells. a) Heatmap of observed C-to-T conversion (%) induced by AncBE4max and 11 AncBE4max variants containing combinate X-to-R mutations at eight endogenous genomic loci in HEK293T cells. The results show the editing efficiency of the all-edited C-to-T within the editing window, data are presented as means (n = 3 biological replicates). The results from all sites were also summarized into a “median” panel placed at the most right-handed position. **b**) On the left, relative indel ratio (%) associated with (a) was shown (means ± s.d., n = 3 biological replicates). On the right, results were further analyzed by considering indel ratio (%) at all sites (n = 8 sites) as a whole. The indel ratio (%) induced by AncBE4max were set as 1.

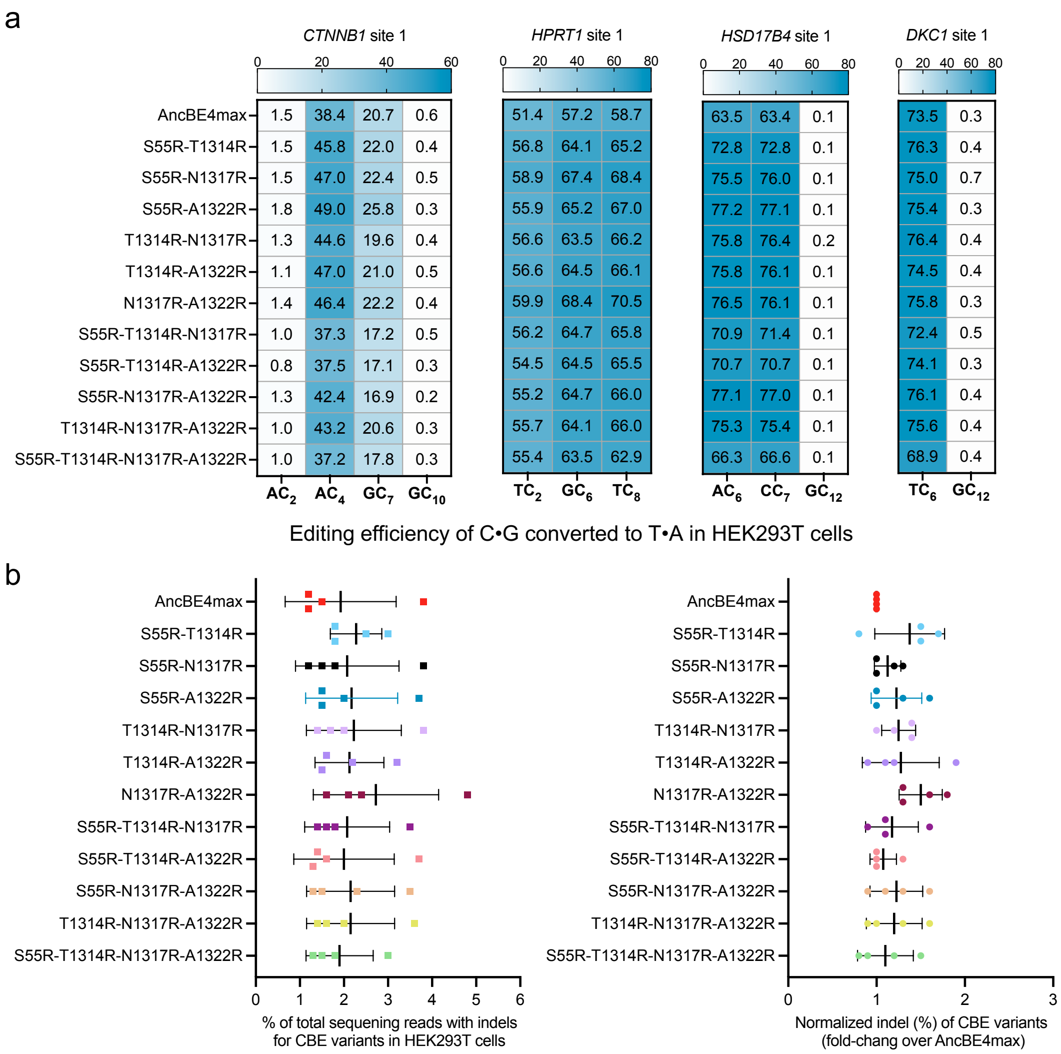

	AC_2	AC_4	GC_7	GC_{10}	TC_2	GC_6	TC_8	AC_6	CC_7	GC_{12}	TC_6	GC_{12}
AncBE4max	1.5	38.4	20.7	0.6	51.4	57.2	58.7	63.5	63.4	0.1	73.5	0.3
S55R-T1314R	1.5	45.8	22.0	0.4	56.8	64.1	65.2	72.8	72.8	0.1	76.3	0.4
S55R-N1317R	1.5	47.0	22.4	0.5	58.9	67.4	68.4	75.5	76.0	0.1	75.0	0.7
S55R-A1322R	1.8	49.0	25.8	0.3	55.9	65.2	67.0	77.2	77.1	0.1	75.4	0.3
T1314R-N1317R	1.3	44.6	19.6	0.4	56.6	63.5	66.2	75.8	76.4	0.2	76.4	0.4
T1314R-A1322R	1.1	47.0	21.0	0.5	56.6	64.5	66.1	75.8	76.1	0.1	74.5	0.4
N1317R-A1322R	1.4	46.4	22.2	0.4	59.9	68.4	70.5	76.5	76.1	0.1	75.8	0.3
S55R-T1314R-N1317R	1.0	37.3	17.2	0.5	56.2	64.7	65.8	70.9	71.4	0.1	72.4	0.5
S55R-T1314R-A1322R	0.8	37.5	17.1	0.3	54.5	64.5	65.5	70.7	70.7	0.1	74.1	0.3
S55R-N1317R-A1322R	1.3	42.4	16.9	0.2	55.2	64.7	66.0	77.1	77.0	0.1	76.1	0.4
T1314R-N1317R-A1322R	1.0	43.2	20.6	0.3	55.7	64.1	66.0	75.3	75.4	0.1	75.6	0.4
S55R-T1314R-N1317R-A1322R	1.0	37.2	17.8	0.3	55.4	63.5	62.9	66.3	66.6	0.1	68.9	0.4

Figure S3. AncBE4max activities are enhanced through the combination of X-to-R mutations in nCas9 at previously examined genomic loci in HEK293T cells. a) Heatmap of observed C-to-T conversion (%) induced by AncBE4max and 11 combinate mutations mediated-AncBE4max variants at four previously examined endogenous genomic loci (detected in Figure 1) in HEK293T cells. The results show the editing efficiency of the all-edited C-to-T within the editing window, data are presented as means (n = 3 biological replicates). **b**) On the left, relative indel ratio (%) associated with (a) was shown (means ± s.d., n = 3 biological replicates). On the right, results were further analyzed by considering indel ratio (%) at all sites (n = 4 sites) as a whole. The indel ratio (%) induced by AncBE4max were set as 1.

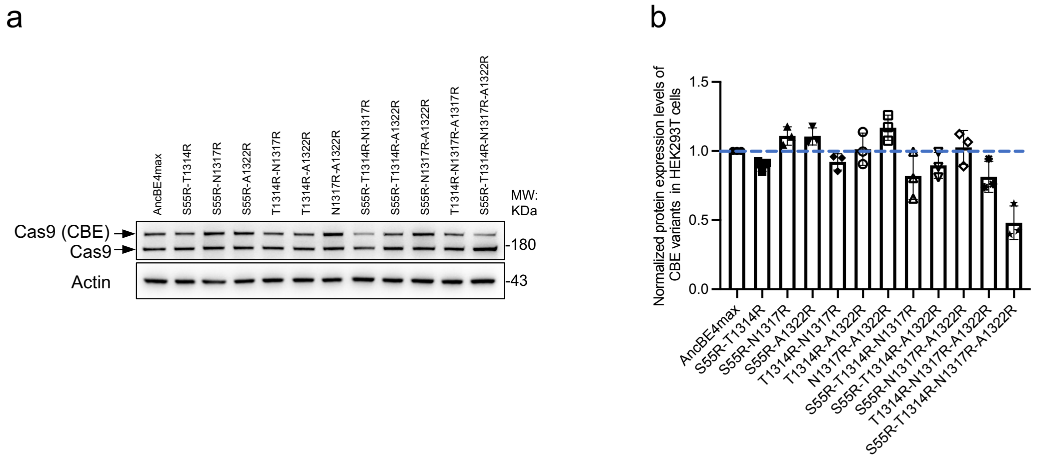

Figure S4. The protein expression levels of AncBE4max variants are comparable in HEK293T cells. a) The protein expression levels of AncBE4max variants were compared, following their transfection in HEK293T cells. The cells were transfected with the same amount of the BE plasmids as that in the earlier editing experiments. In addition, an equal amount of WT Cas9 plasmid was co-transfected with the AncBE4max variants, to serve as an internal control. A representative result from three independent experiments is shown here. The Western blot analysis indicated that the average level of the 2XR group was close to the control nCas9/AncBE4max, and most 3XR variants and the 4XR variant showed comparatively lower expression (4XR at the lowest). **b**) The Western blots were scanned for further quantitation. The levels of the control AncBE4max was set as 1.

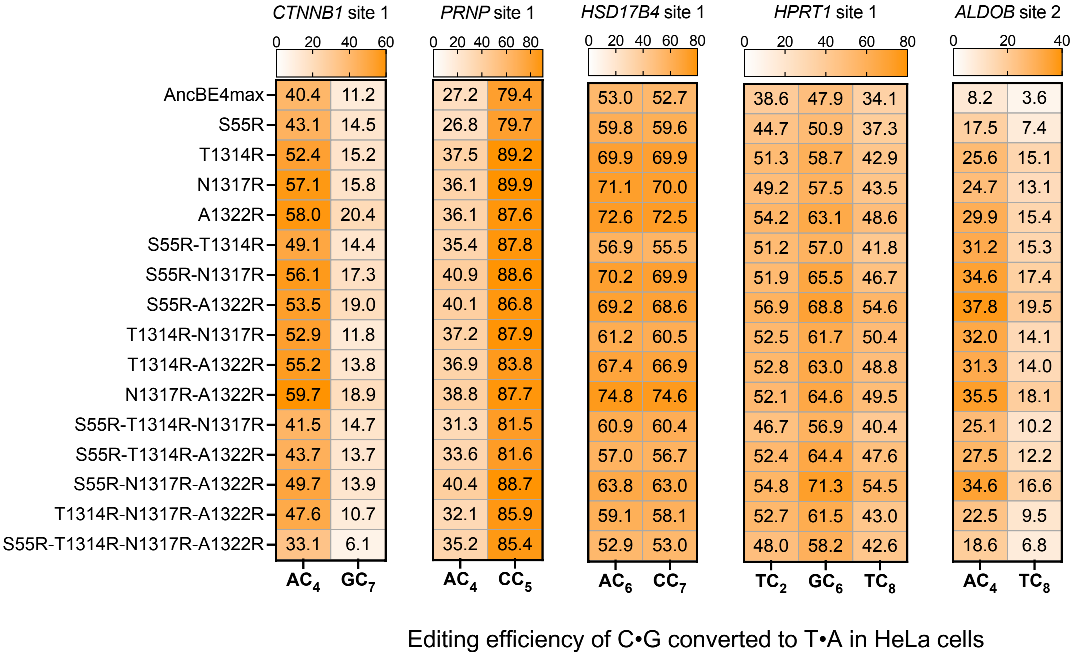

Figure S5. Combination of X-to-R mutations in nCas9 for further improvement of AncBE4max activities at more tested sites in HeLa cells. Heatmap of observed C-to-T conversion (%) induced by AncBE4max and 11 combinate mutations mediated-AncBE4max variants at five endogenous genomic loci in HeLa cells. The results show the editing efficiency of the all-edited C-to-T within the editing window, data are presented as means (n = 3 biological replicates).

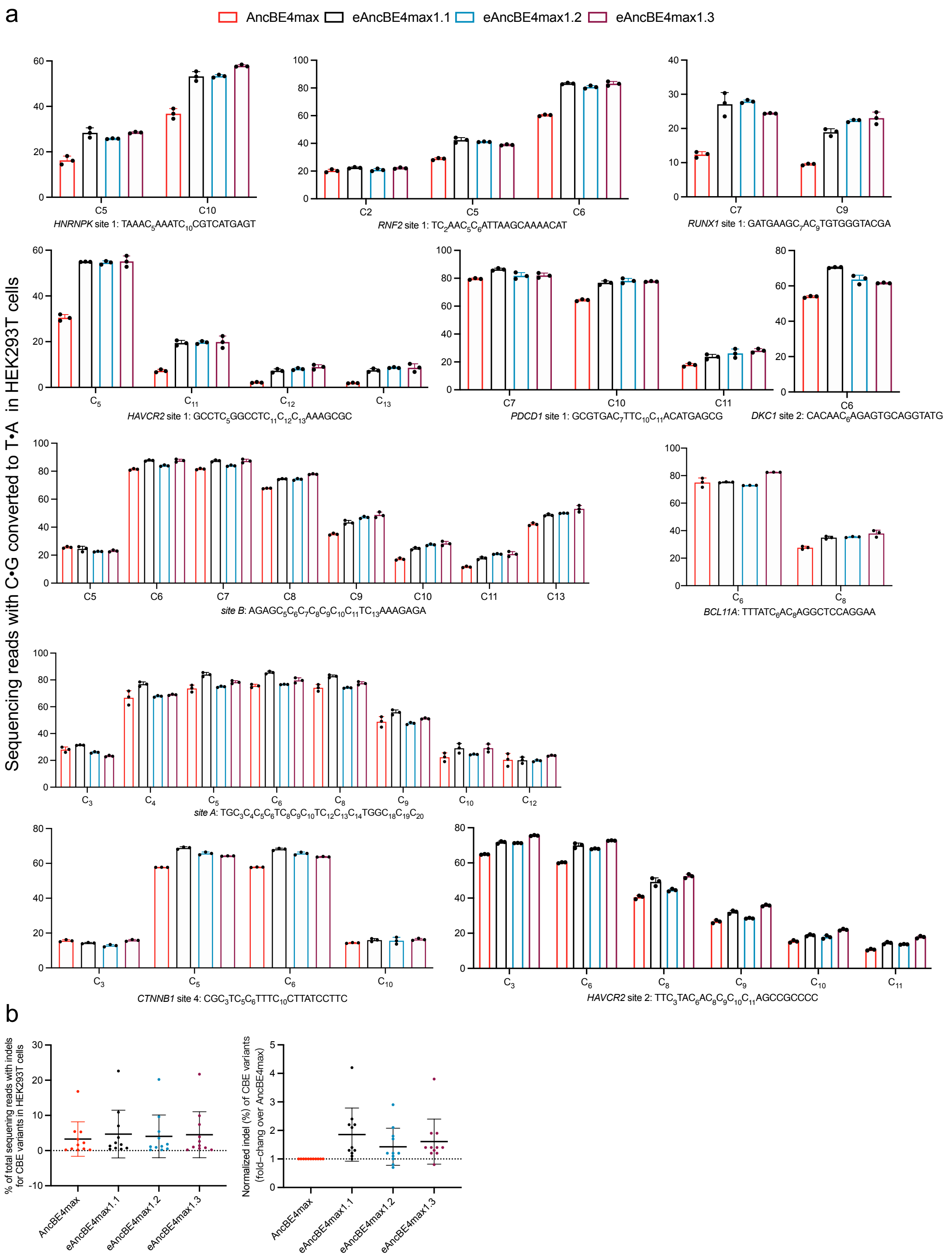

Figure S6. Compare the editing efficiency of AncBE4max and eAncBE4max1.1~1.3 in HEK293T cells. a) The editing efficiency of AncBE4max and eAncBE4max1.1~1.3 at eleven genomic loci in HEK293T cells was analyzed by NGS, data are presented as means ± s.d. (n = 3 biological replicates). **b**) Relative indel ratio (%) of AncBE4max and eAncBE4max1.1~1.3 associated with (a) are shown (means ± s.d., n = 3 biological replicates). The figure on the right, results were further analyzed

by considering indel ratio (%) at all sites (n = 11 sites) as a whole. The indel ratio (%) induced by AncBE4max were set as 1.

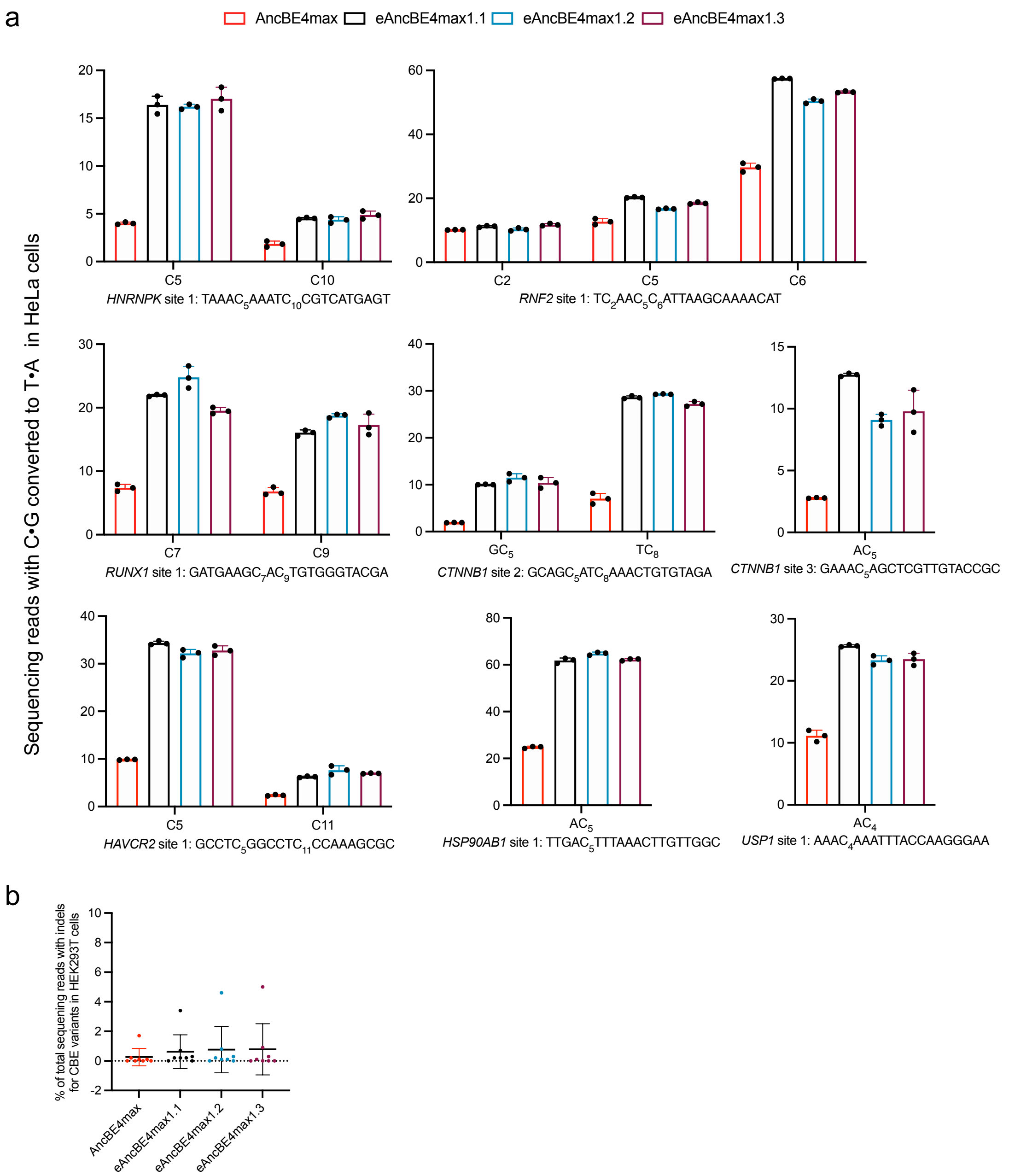

Figure S7. Compare the editing efficiency of AncBE4max and eAncBE4max1.1~1.3 in HeLa cells. a) The editing efficiency of AncBE4max and three top-performing double mutants at eight genomic loci in HeLa cells, data are presented as means ± s.d. (n = 3 biological replicates). **b**) Relative indel ratio (%) associated with (a) (means ± s.d., n = 3 biological replicates).

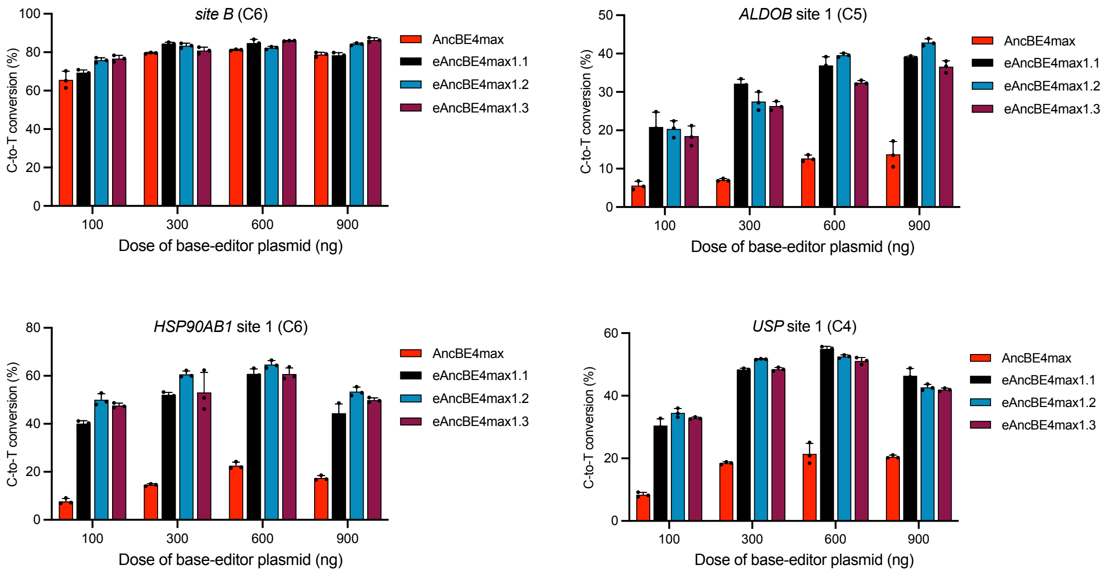

Figure S8. The eAncBE4max-s apparently exhibited higher activities over the control AncBE4max at all editor doses tested. Compared the editing efficiency of AncBE4max and eAncBE4max1.1~1.3 at four genomic loci in HEK293T cells across four editor plasmid doses (means ± s.d., n = 3 biological replicates).

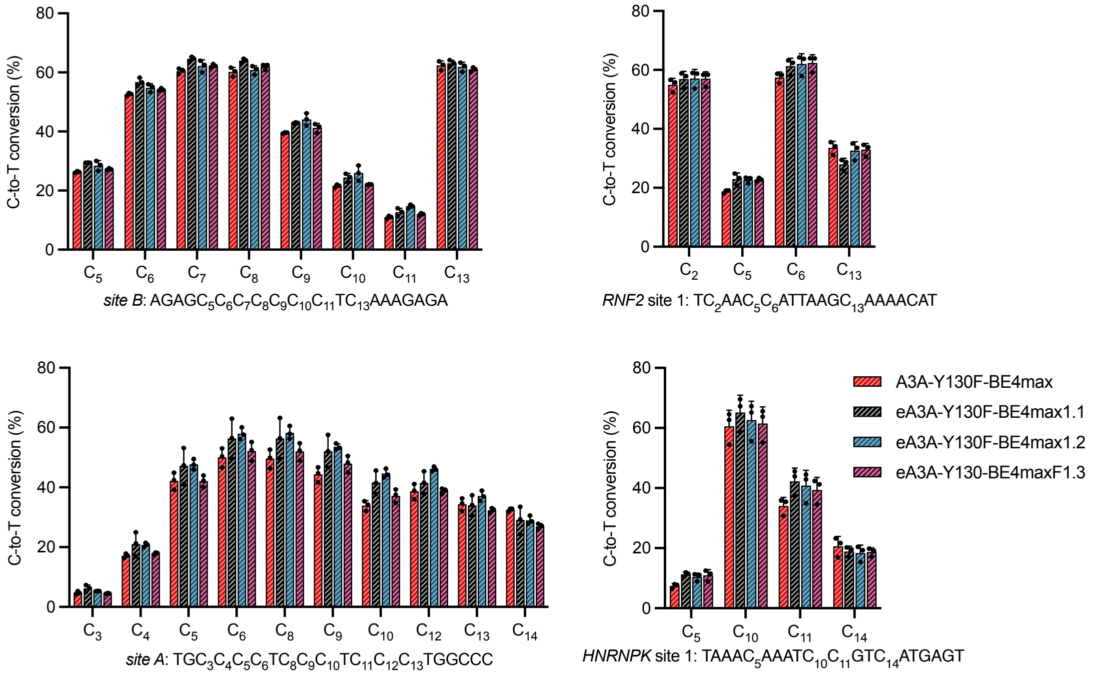

Figure S9. At those sites where the A3A-Y130F already exhibited high editing rates, the Xs-to-Rs-modified nCas9 did little to further enhance the performances. Evaluated the C-to-T conversion efficiency (%) in HEK293T cells by A3A-Y130F-BE4max (created by substituting Anc689 deaminase in AncBE4max with A3A-Y130F) and eA3A-Y130F-BE4max1.1~1.3 (consisting of the double mutants S55R-N1317R, S55R-A1322R, and N1317R-A1322R in A3A-Y130F-BE4max, labeled as eA3A-Y130F-BE4max1.1, eA3A-Y130F-BE4max1.2, and eA3A-Y130F-BE4max1.3 respectively), at four specified genomic sites. data are presented as means ± s.d. (n = 3 biological replicates).

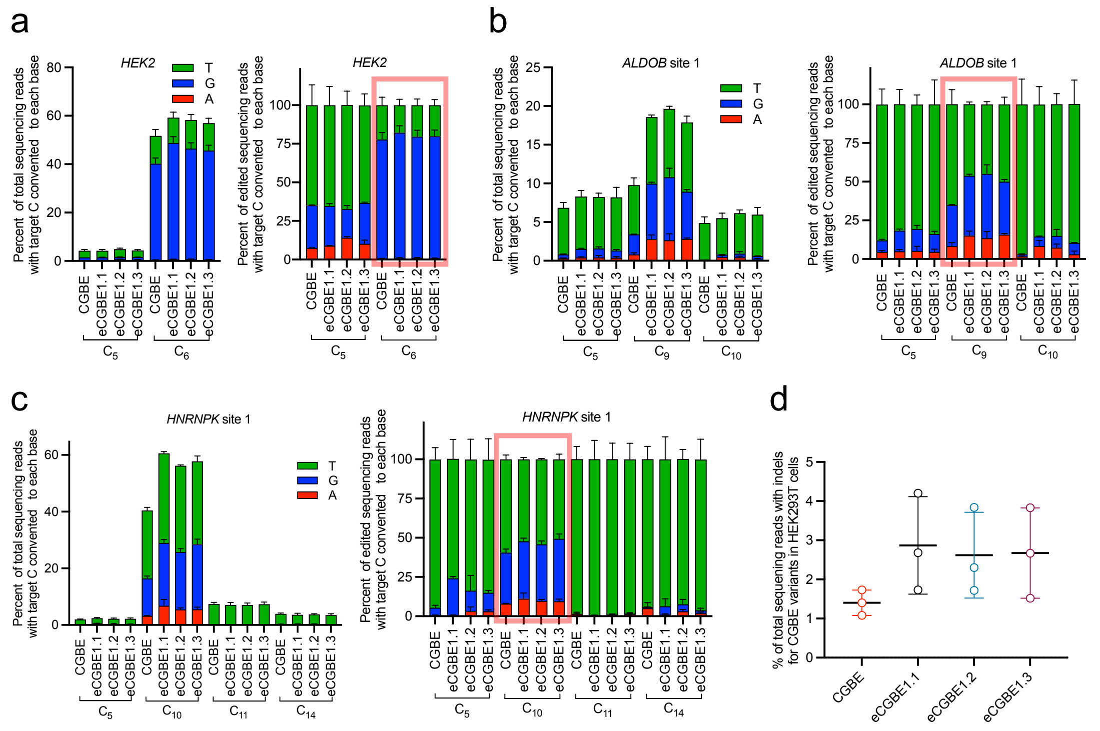

Figure S10. Optimize nCas9 for increasing the editing efficiency of CGBE in HEK293T cells. a) HEK293T cells were transfected with CGBE (UdgX-Anc689-UdgX-nCas9-RBMX), and eCGBE1.1~1.3 (we defined S55R-N1317R, S55R-A1322R and N1317R-A1322R double mutants mediated-CGBE as eCGBE1.1, eCGBE1.2 and eCGBE1.3, respectively), as described in Experimental Section. On the left, the percent of total sequencing reads with target C convened to each base at *HEK2* site were shown. On the right, the distribution of C convened to each base among edited DNA sequencing reads (reads in which the target C were mutated) were shown. The boxed region corresponds to the most susceptible base. **b**) Same experiment as (a), the editing situation of CGBEs at *ALDOB* site 1 were shown. **c**) Same experiment as (a), the editing situation of CGBEs at *HNRNPK* site 1 were shown. **d**) Relative indel ratio (%) associated with Figure 4d and Figure 10a-c, Supporting Information were shown (means ± s.d., n = 3 biological replicates).

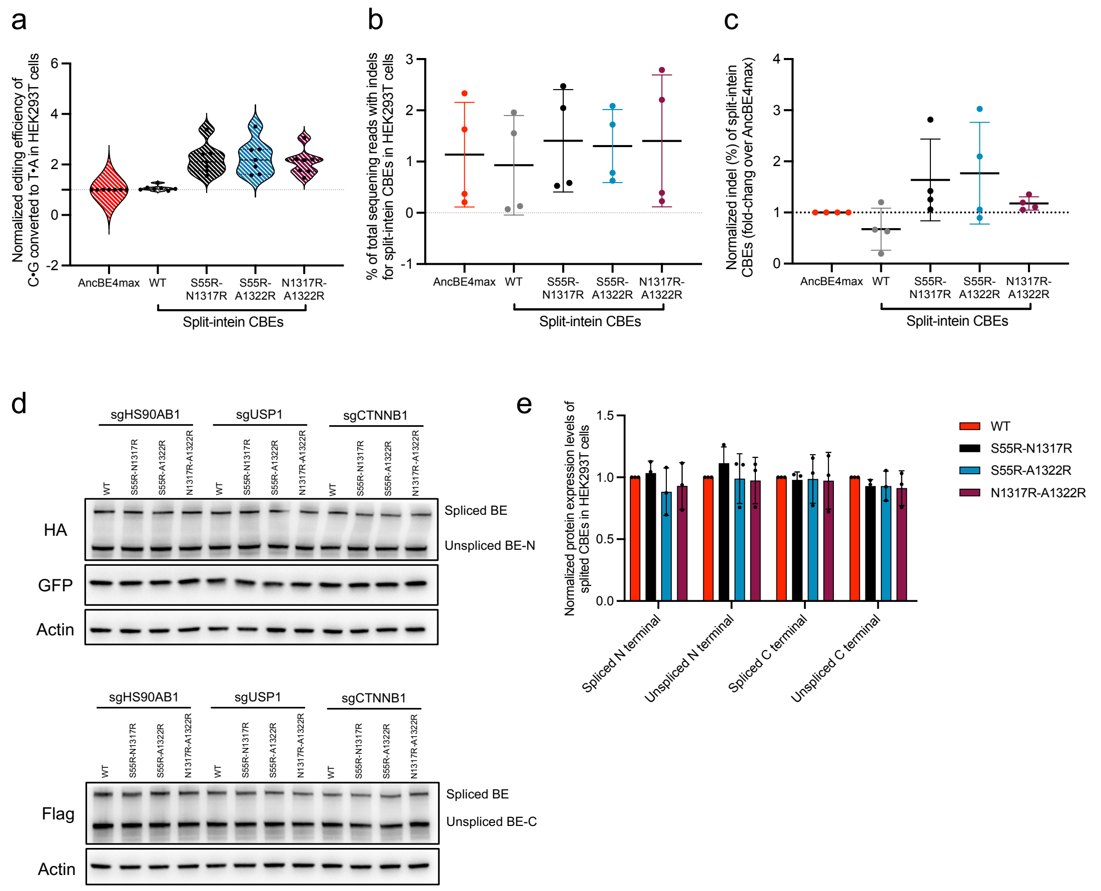

Figure S11. Split eAncBE4max-s increase editing efficiency without compromising fidelity, and their protein expression levels are comparable to split AncBE4max in HEK293T cells. a) C-to-T conversion (%) induced by AncBE4max, split-AncBE4max (split-WT) and the S55R-N1317R-, S55R-A1322R- and N1317R-A1322R-introduced split-BEs at four target contexts in HEK293T cells are analyzed by considering editing efficiency at all sites (n = 4 sites) as a whole. The editing frequencies induced by AncBE4max were set as 1 (gray dashed line). **b**) HEK293T cells were transfected with AncBE4max and split-BEs (including split AncBE4max and split eAncBE4max-s). The indel rates (%) induced by AncBE4max and split-BEs at four genomic sites were considered as a whole (n = 4 sites). **c**) Results in (b) were normalized. The indel rate (%) induced by AncBE4max were set as 1 (gray dashed line). **d**) The split BEs were tagged at the N- and C-terminus with HA and Flag, respectively. The protein expression levels of split AncBE4max and eAncBE4max-s were determined, following their transfections in HEK293T cells under the same condition as the earlier editing experiments. Three different sgRNA plasmids were also co-transfected. The Western

blot analysis indicated that the average levels of the split and full-length parts of the 2XR groups were comparable to those of the control split AncBE4max group. **e**) Results in (d) were quantitated. The expression level of the split AncBE4max was set as 1.

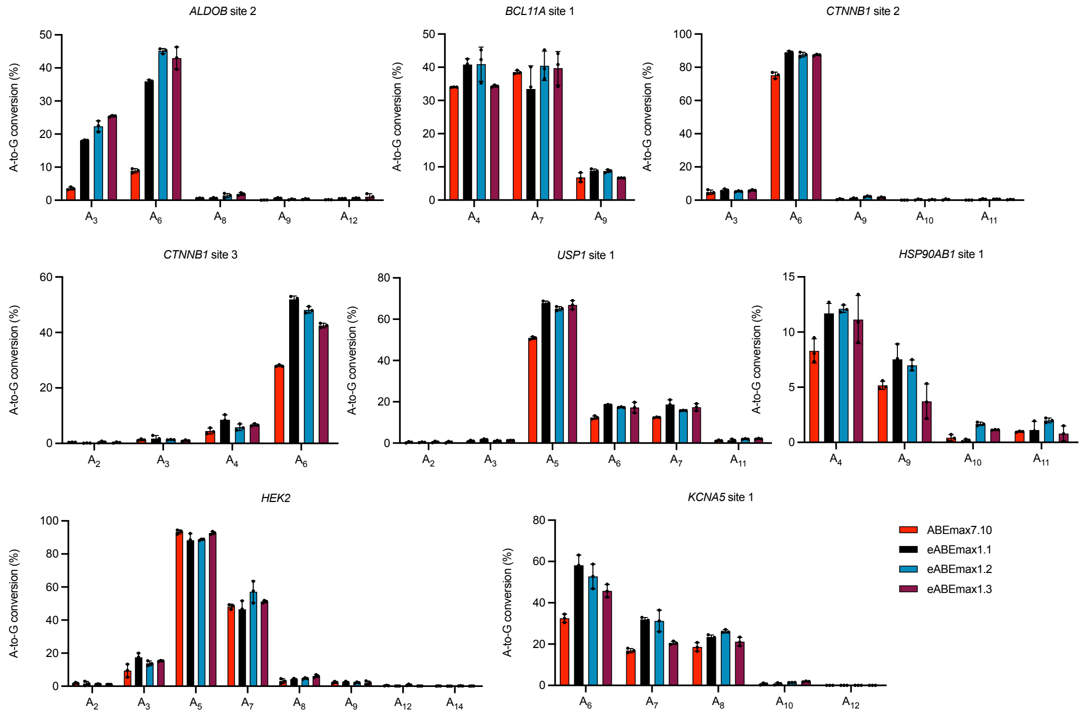

Figure S12. Similarly engineered (with the favorable 2XR modifications) eABEmax1.1~1.3 variants could generally enhance the activities of ABEmax7.10 in HEK293T cells. The editing efficiency of ABEmax7.10 and eABEmax1.1~1.3 at eight genomic loci in HEK293T cells are shown. Data are presented as means ± s.d. (n = 3 biological replicates).

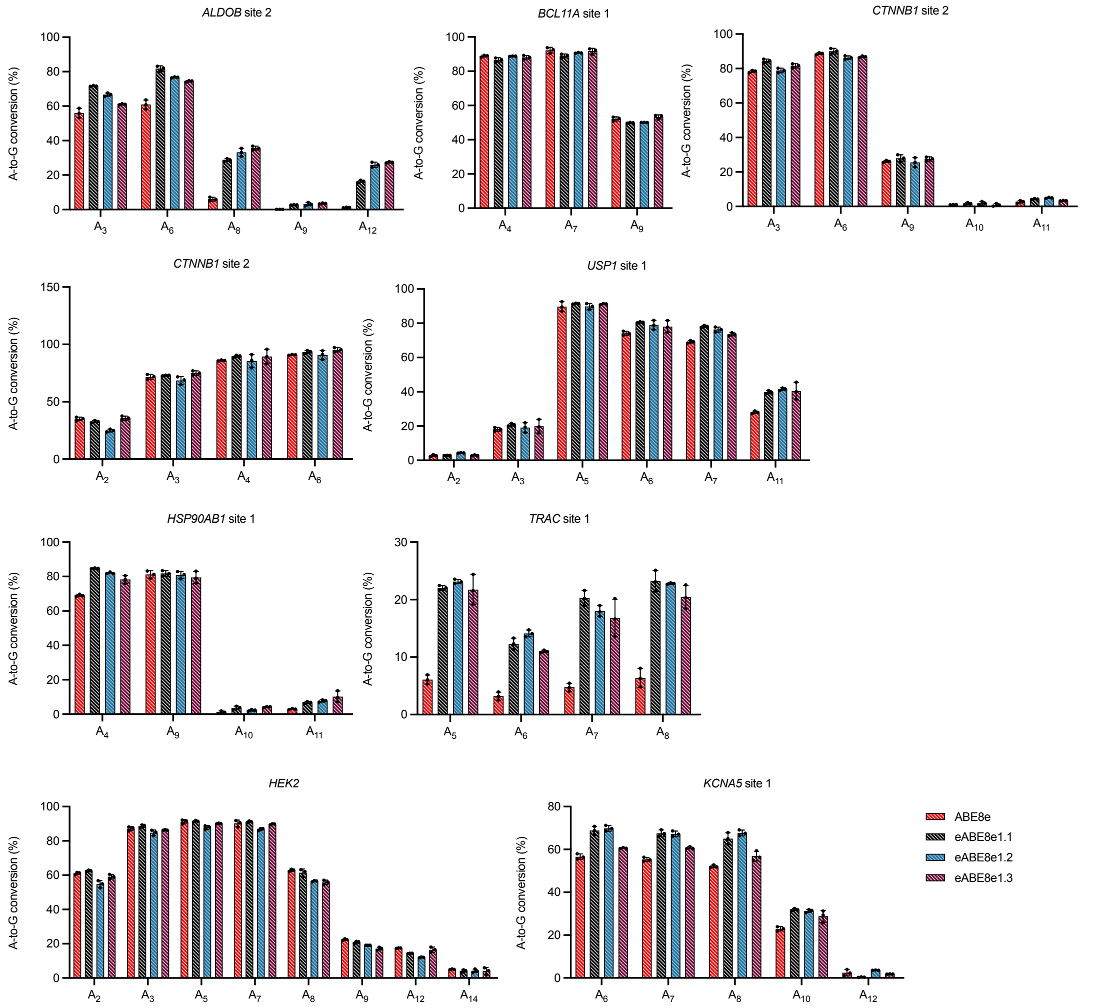

Figure S13. The eABE8e-1.1~1.3 variants showed conditional enhancement effects mainly at sites less susceptible to ABE8e in HEK293T cells. The editing efficiency of ABE8e and eABE8e-1.1~1.3 at nine genomic loci in HEK293T cells are shown, data are presented as means ± s.d. (n = 3 biological replicates).

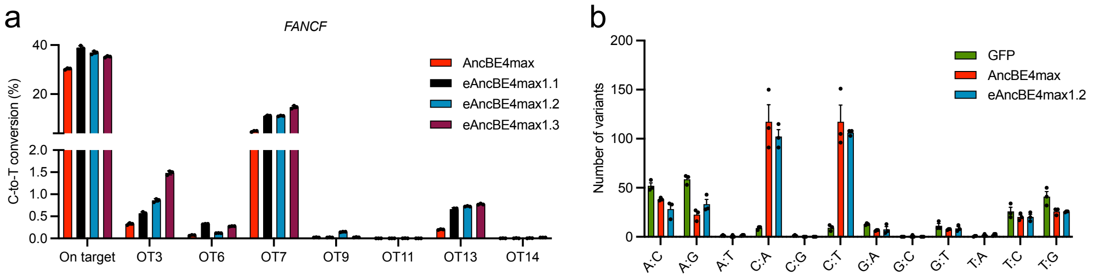

Figure S14. Assessments of the DNA or RNA off-target editing activity of eAncBE4max-s in HEK293T cells. a) HEK293T cells were transfected with AncBE4max and eAncBE4max (1.1~1.3) plasmids together with a sgRNA. The levels of editing at the on- and off-target sites for the indicated sgRNA (against *FANCF*) are shown (mean ± s.d., n = 3 biological replicates). **b**) Comparison of off-target RNA editing activity by AncBE4max and eAncBE4max1.2.

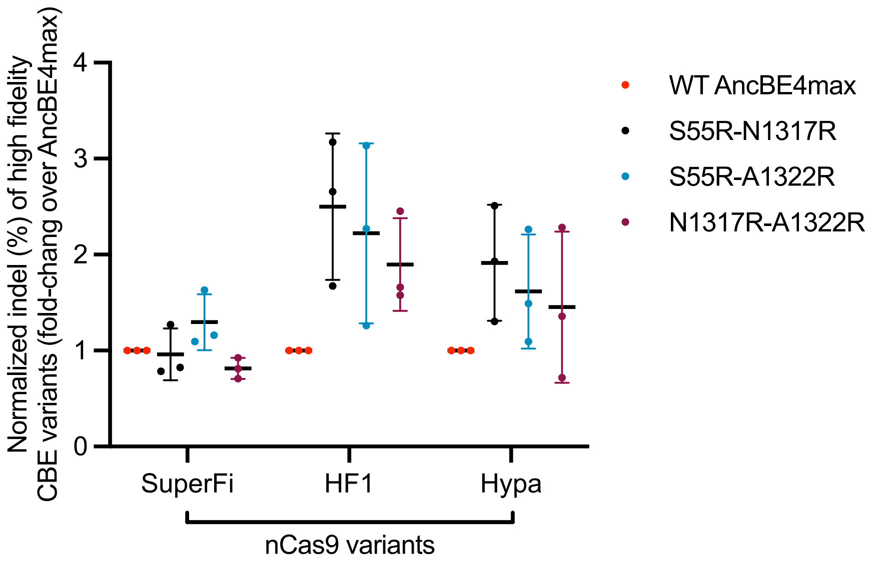

Figure S15. The indel rates (%) induced by high-fidelity CBEs and WT CBE are comparable in HEK293T cells. We constructed high-fidelity versions of the 2XR-engineered BEs by merging the 2XR substitutions and SuperFi, HF1 and Hypa nCas9. Indel rates (%) induced by high-fidelity versions of BE and engineered BE variants at three target sites in HEK293T cells were considered as a whole. The indel ratio (%) induced by the original high-fidelity AncBE4max forms (with the original SuperFi, HF1 and Hypa nCas9) were set as 1. Data are presented as means ± s.d. (n = 3 biological replicates).

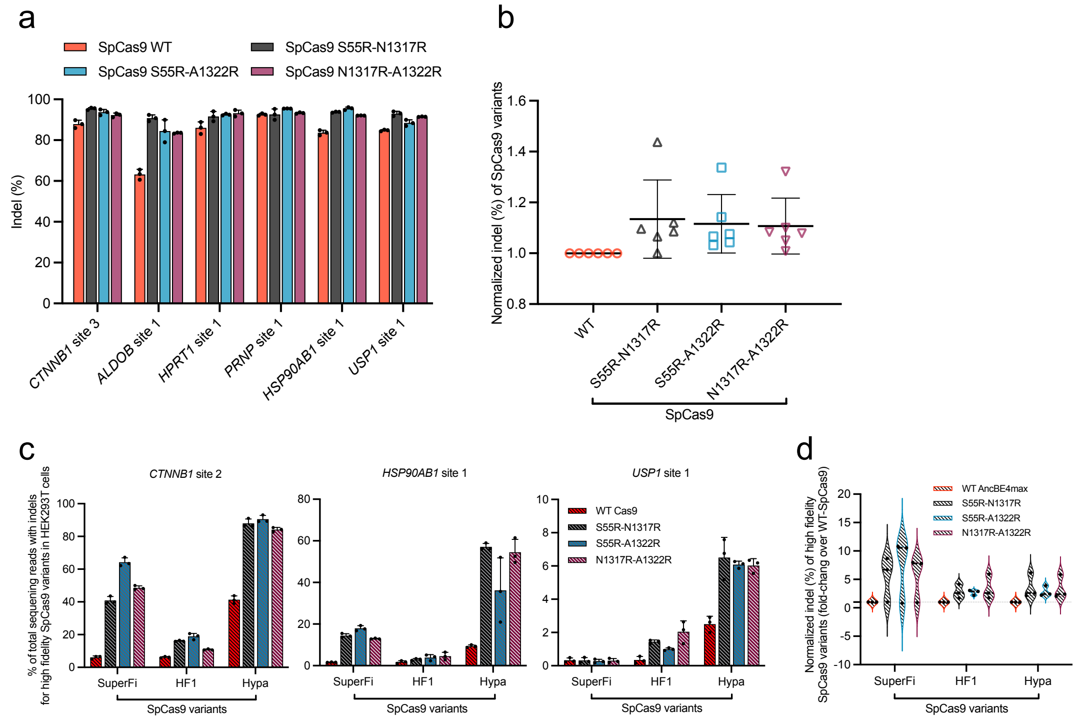

Figure S16. 2XR modifications in Cas9 and HF-Cas9 caused differential levels of increases in their cleaving efficiencies. a) HEK293T cells were transfected with WT SpCas9 or the Xs-to-Rs-modified Cas9 variants (containing the double mutants S55R-N1317R, S55R-A1322R and N1317R-A1322R) together with sgRNAs. The indel rates (%) of WT SpCas9 or the Cas9 variants at six genomic sites were shown (mean ± s.d., n = 3 biological replicates). **b**) Results in (a) were further analyzed by considering indel rates (%) at all sites (n = 6 sites) as a whole. The indel rates (%) induced by WT SpCas9 were set as 1. **c**) HEK293T cells were transfected with WT HF-Cas9 or HF-Cas9 variants (SuperFi, HF1 and Hypa versions, containing the double mutants S55R-N1317R, S55R-A1322R and N1317R-A1322R) together with sgRNA. The indel (%) of WT HF-Cas9 or HF-Cas9 variants at three genomic sites were shown (mean ± s.d., n = 3 biological replicates). **d)** Results in (c) were further analyzed by considering indel rates (%) at all sites (n = 3 sites) as a whole. The indel rates (%) induced by WT HF-Cas9 were set as 1.

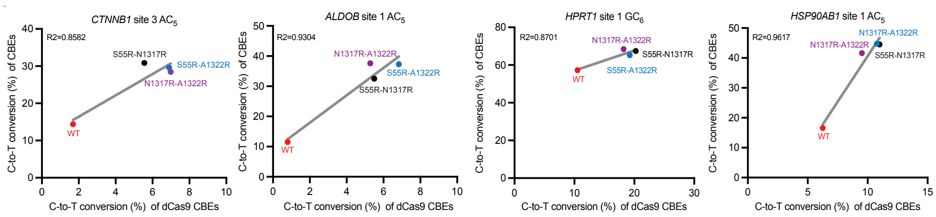

Figure S17. The correlation between editing efficiencies of nCas9- and dCas9-based AncBE4max variants. The correlations between the editing efficiencies of dead Cas9-based AncBE4max (data in Figure 6c), and nCas9-based AncBE4max (data in Figure S2a and Figure S3a, Supporting Information) at four target sites are presented.

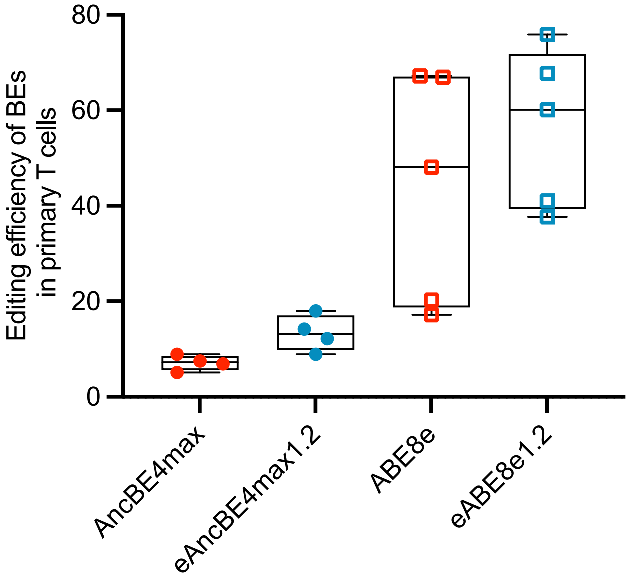

Figure S18. Overall efficiencies by engineered AncBE4max and ABE8e in primary human T cells. The control and engineered BEs (in the forms of AncBE4max or ABE8e) were adopted to edit relevant targets in human T cells (see detailed data in Figure 7). LNP vector was used for delivering the BEs to primary human T cells. The overall efficiencies of AncBE4max-s (control and engineered form 1.2) at 4 target cytosines, and of ABE8e-s (control and engineered form 1.2) at the substrate adenines from 5 targeted splicing sites in human T cells are summarized. The center line shows medians of all data points and the box limits correspond to the upper the lower quartiles, while the whiskers extend to the largest and smallest values.

Table S1. Primers used for engineered nCas9 variants construction.

NO.	Forward primer	Reverse primer
R780A	GCGAGGCAATGAAGCGGATCGAAGAGGGC	GCTTCATTGCCTCGCGGCTGTTCTTCTGTC
R783A	TGAAGGCAATCGAAGAGGGCATCAAAGAGC	CTTCGATTGCCTTCATTCTCTCGCGGCTGTTC
K810A	ACGAGGCACTGTACCTGTACTACCTGCAG	GGTACAGTGCCTCGTTCTGCAGCTGGGTGTTTTCCA
R832A	CATCAACGCACTGTCCGACTACGATGTGGA	GTTGATGTCCAGTTCCTGGT
R848A	TTTCTGGCAGACGACTCCATCGACAACAAG	GTCGTCTGCCAGAAAGCTCTGAGGCACGATATG
K855A	ACAACGCAGTGCTGACCAGAAGCGACAAGAAC	TCAGCACTGCGTTGTCGATGGAGTCGTCCTTC
R859A	CTGACCGCAAGCGACAAGAACCGGGGCAAG	GTCGCTTGCGGTCAGCACCTTGTTGTCGATG
S964A	CTGGTGGCAGATTTCCGGAAGGATTTCCA	GAAATCTGCCACCAGCTTGGACTTCAGGGTG
K968A	TTCCGGGCAGATTTCCAGTTTTACAAAGTGCG	GAAATCTGCCCGGAAATCGGACACCAGCTTG
R976A	AAAGTGGCAGAGATCAACAACTACCACCAC	GATCTCTGCCACTTTGTAAAACTGGAAATCCTTC
H982A	AACTACGCACACGCCCACGACGCCTACCTAAACG	GGCGTGTGCGTAGTTGTTGATCTCGCGCACTTTG
K1003A	TACCCTGCACTGGAAAGCGAGTTCGTGTACG	TTCCAGTGCAGGGTACTTTTTGATCAGGGC
K1047A	TTTTTCGCAACCGAGATTACCCTGGCCAACGGC	CTCGGTTGCGAAAAAGTTCATGATGTTGCTG
R1060A	GGCGAGATCCGGAAGGCACCTCTGATCGAGACAAACGGCGAAACCGGGGAGATCGTGTG	CTTCCGGATCTCGCCGTTGGCCAGGGTAATCTCGGTCTTGAAAAAGTTCATGATGTTGC
D54R	TTCCGTAGCGGCGAAACAGCCGAGGCCACCCGGCTGAAG	TTCGCCGCTACGGAACAGCAGGGCTCCGATCAGGTTCTTCTTGATGC
S55R	TTCGACCGCGGCGAAACAGCCGAGGCCACCCGGCTGAAGA	TTCGCCGCGGTCGAACAGCAGGGCTCCGATCAGGTTCTTCTTGATGC
N980R	ATCAACCGCTACCACCACGCCCACGACGCCTACCTAAACG	GTGGTAGCGGTTGATCTCGCGCACTTTGTAAAACTGGAAATCCTTCCGGAAATCGGACA
T1314R	TTCGCCTGACCAATCTGGGAGCCCCTGCCGCCTTCAAG	GATTGGTCAGGCGAAACAGGTGGATGATATTCTCGGCCTGCTCTCTGA
N1317R	TGACCCGCCTGGGAGCCCCTGCCGCCTTCAAGTACTTTGACA	CTCCCAGGCGGGTCAGGGTAAACAGGTGGATGATATTCTC
A1322R	TCGCGCCTTCAAGTACTTTGACACCACCATCGACCGGA	TACTTGAAGGCGCGAGGGGCTCCCAGATTGGTCAGGGTAAACAGGTGGA

Table S2. Spacer sequences used for constructing sgRNA plasmids.

NO.	Forward primer	Reverse primer
ALDOB site 1	ACCGCATACTGTCCTTTGGCCGCC	AAACGGCGGCCAAAGGACAGTATG
HSP90AB1 site 1	ACCGTTGACTTTAAACTTGTTGGC	AAACGCCAACAAGTTTAAAGTCAA
CTNNB1 site 1	ACCGACACAGCAGCAATTTGTGGT	AAACACCACAAATTGCTGCTGTGT
HPRT1 site 1	ACCGTCTTGCTCGAGATGTGATGA	AAACTCATCACATCTCGAGCAAGA
HSD17B4 site 1	ACCGGTGTACCAAGGCCCTGCAAA	AAACTTTGCAGGGCCTTGGTACAC
DKC1 site 1	ACCGGTGGTCAGATGCAGGAGCTT	AAACAAGCTCCTGCATCTGACCAC
USP1 site 1	ACCGAAACAAATTTACCAAGGGAA	AAACTTCCCTTGGTAAATTTGTTT
ALDOB site 2	ACCGCTACTAGAAGCACTGGAGCT	AAACAGCTCCAGTGCTTCTAGTAGG
PRNP site 1	ACCGGAACCTTGGCTGCTGGATGC	AAACGCATCCAGCAGCCAAGGTTC
FANCF site 1	ACCGCCAGCAGGCGCAGAGAGAGC	AAACGCTCTCTCTGCGCCTGCTGG
CTNNB1 site 2	ACCGGCAGCATCAAACTGTGTAGA	AAACTCTACACAGTTTGATGCTGC
CTNNB1 site 3	ACCGGAAACAGCTCGTTGTACCGC	AAACGCGGTACAACGAGCTGTTTC
HNRNPK site 1	ACCGTAAACAAATCCGTCATGAGT	AAACACTCATGACGGATTTGTTTA
RNF2 site 1	ACCGTCAACCATTAAGCAAAACAT	AAACATGTTTTGCTTAATGGTTGA
RUNX1 site 1	ACCGGATGAAGCACTGTGGGTACGA	AAACTCGTACCCACAGTGCTTCATC
HAVCR2 site 1	ACCGGCCTCGGCCTCCCAAAGCGC	AAACGCGCTTTGGGAGGCCGAGGC
HAVCR2 site 2	ACCGTTCTACACCCCAGCCGCCCC	AAACGGGGCGGCTGGGGTGTAGAA
PDCD1 site 1	ACCGGCGTGACTTCCACATGAGCG	AAACCGCTCATGTGGAAGTCACGC
DKC1 site 2	ACCGACCGCAGACTCCCGCCGCCT	AAACAGGCGGCGGGAGTCTGCGGT
site B	ACCGAGAGCCCCCCCTCAAAGAGA	AAACTCTCTTTGAGGGGGGGCTCT
site A	ACCGTGCCCCTCCCTCCCTGGCCC	AAACGGGCCAGGGAGGGAGGGGCA
BCL11A site 1	ACCGTTTATCACAGGCTCCAGGAA	AAACTTCCTGGAGCCTGTGATAAA
FANCF	ACCGGGAATCCCTTCTGCAGCACC	AAACGGTGCTGCAGAAGGGATTCC
HEK2	ACCGGAACACAAAGCATAGACTGC	AAACGCAGTCTATGCTTTGTGTTC
KCNA5	ACCGCCGGGAAACAGATTTGTGAA	AAACTTCACAAATCTGTTTCCCGG
TRAC	ACCGTTTCAAAACCTGTCAGTGAT	AAACATCACTGACAGGTTTTGAAA

Table S3. Primers used for HEK293T and HeLa cells genomic DNA amplification and targeted deep sequencing.

NO.	Forward primer	Reverse primer
ALDOB site 1	CTCCTACTAGAAGCACTGGAG	CTGAGTGAAGGTTTGACTGG
HSP90AB1 site 1	GGTATTGCAGTTCTGTAGGC	ACAGTGAAGGAACCTCCAGC
CTNNB1 site 1	TCATGCACCTTTGCGTGAGC	TCTCTGCAGCCATAGAAATG
HPRT1 site 1	CACTATATTGCCCAGGTTGGTGTGG	GATAAAATCTACAGTCATAGGAATG
HSD17B4 site 1	ATGTGTAGAATAAGTGCCAC	TGAGGGTCAAGCTTGCCCAG
DKC1 site 1	GAGGCTGGCACCTACATTCG	AACTCAACACTTTGGAAAGC
USP1 site 1	TTCAAATCCCAAGGAGTGTC	TTAAGAACAGTGTGGTATGC
ALDOB site 2	GTCACATTTACTCTAACCAG	ACATGTGTTGTATTTCCAGC
PRNP site 1	TGAGCAGCTGATACCATTGC	GCGGTTGCCTCCAGGGCTGC
FANCF site 1	CCTGCGCCACATCCATCGGC	TGCACCAGGTGGTAACGAGC
CTNNB1 site 2	GTGGCAAGTTCTGCATCATC	GCTGAACTGTGGATAGTGAG
CTNNB1 site 3	TGCAGTTATGGTCCATCAGC	TTAGCTTCAAGCATTCTGAC
HNRNPK site 1	CATGCTCCTTGAAAATACAG	CTTAAACTGACCTGTTCTGC
RNF2 site 1	AGCCAACATACAGAAGTCAG	TTTCCAGCAATGTCTCAGGC
RUNX1 site 1	AAGAAAGAGAGATGTAGGGC	CATTACAGGCAAAGCTGAGC
HAVCR2 site 1	GTAGCTGGGATTACAGACGC	TTGAGAAGCTCAGAGGTTCG
HAVCR2 site 2	TGGAGTAACCTCACTCACCG	TTAGCCAGTATCTGGATGTC
PDCD1 site 1	AACCAGACGGACAAGCTGGC	ACCTGTCACCCTGAGCTCTG
DKC1 site 2	AATCGCATTGCGCAGACGAC	GAGTTAGCACGGCCCGGAAG
site B	CTTCACTGAGTCTCCACACA	GAAATCTTAGGAACTGAGAG
site A	CTAACCCTATGTAGCCTCAG	AATGCGCCACCGGTTGATGT
BCL11A site 1	CAGGTAATAACATAGGCCAG	CAAGAGAGCCTTCCGAAAGA
FANCF	AAAGACGCTGGGAGATTGAC	CCAGGTGCTGACGTAGGTAG
HEK2	TCAAGTTACTGCAGCCCAAG	CCCCATCTGTCAAACTGTGC
KCNA5	GAGAAGTGTAACGTCAAGGC	GAGGGGTAGACTGAGGTTAC
TRAC	AAACCGTGGGTGTGTCCTGC	GCTCTCAGAGCTTAGGATGC

Table S4. Spacer sequences of sgRNAs used for primary T cell editing.

NO.	Sequences (5'-3')
B2M iSTOP	TTACCCCACTTAACTATCTT
CD247 iSTOP	CAGGCACAGTTGCCGATTAC
CD3D iSTOP	TCTATCAGGTGAGCGTTGAG
CTLA4 site 1	CACTCACCTTTGCAGAAGAC
CTLA4 site 2	GCTCACCAATTACATAAATC
PDCD1 sgRNA1	CACCTACCTAAGAACCATCC
PDCD1 sgRNA2	ATCTCTCAGACTCCCCAGAC
B2M sgRNA1	CTTACCCCACTTAACTATCT

Table S5. Primers used for primary T cells genomic DNA amplification and targeted deep sequencing.

NO.	Forward primer	Reverse primer
B2M iSTOP	AGATGGGATGGGACTCATTC	CTATCTCTTGTACTACACTG
CD247 iSTOP	GACTCCTTTTCTCCTAACCG	GAGGGCAGGATTTGAAGGAG
CD3D iSTOP	CTCACAGTCCCATCTGCTAG	ACCTCTCCAGTCACACCCAG
CTLA4 site 1	CTGCCTTTGACTGCTGAAAC	TACTTCCTGAAGACCTGAAC
CTLA4 site 2	TGAGTTGACCTTCCTAGATG	ACCTCCTGAAATTAAGGAAC
PDCD1 sgRNA1	CTGGCTCTGGGACACCTGAC	CCGCCTGAGCAGTGGAGAAG
PDCD1 sgRNA2	CTTCTCAATGACATTCCAGC	CTCCGATGTGTTGGAGAAGC
B2M sgRNA1	AGATGGGATGGGACTCATTC	CTATCTCTTGTACTACACTG
